# The Swipe Card Model of Odorant Recognition

**DOI:** 10.3390/s121115709

**Published:** 2012-11-12

**Authors:** Jennifer C. Brookes, Andrew P. Horsfield, A. Marshall Stoneham

**Affiliations:** 1 Department of Chemistry and Chemical Biology, Harvard University, Oxford Street, Cambridge, MA 02138, USA; 2 Department of Materials, Imperial College London, South Kensington Campus, London SW7 2AZ, UK; 3 London Centre for Nanotechnology, 17-19 Gordon Street, London WC1H 0AH, UK; E-Mail: a.stoneham@ucl.ac.uk

**Keywords:** odorant, tunnelling, phonon, detection, activation

## Abstract

Just how we discriminate between the different odours we encounter is not completely understood yet. While obviously a matter involving biology, the core issue is a matter for physics: what microscopic interactions enable the receptors in our noses-small protein switches—to distinguish scent molecules? We survey what is and is not known about the physical processes that take place when we smell things, highlighting the difficulties in developing a full understanding of the mechanics of odorant recognition. The main current theories, discussed here, fall into two major groups. One class emphasises the scent molecule's shape, and is described informally as a “lock and key” mechanism. But there is another category, which we focus on and which we call “swipe card” theories: the molecular shape must be good enough, but the information that identifies the smell involves other factors. One clearly-defined “swipe card” mechanism that we discuss here is Turin's theory, in which inelastic electron tunnelling is used to discern olfactant vibration frequencies. This theory is explicitly quantal, since it requires the molecular vibrations to take in or give out energy only in discrete quanta. These ideas lead to obvious experimental tests and challenges. We describe the current theory in a form that takes into account molecular shape as well as olfactant vibrations. It emerges that this theory can explain many observations hard to reconcile in other ways. There are still some important gaps in a comprehensive physics-based description of the central steps in odorant recognition. We also discuss how far these ideas carry over to analogous processes involving other small biomolecules, like hormones, steroids and neurotransmitters. We conclude with a discussion of possible quantum behaviours in biology more generally, the case of olfaction being just one example. This paper is presented in honour of Prof. Marshall Stoneham who passed away unexpectedly during its writing.

## 1. Introduction

### 1.1. A Brief Introduction to Our Senses

Our senses allow us to demystify our surroundings. It is surprising then perhaps that one of our senses (smell) is still somewhat mysterious. Our senses receive and record input from the environment in order for us to respond and react in a fashion conducive to survival. A type of sense can vary from the very basic chemotaxis that plants exhibit as they grow towards light to the quite complex issue of pheremonal signalling in mate selection. Senses and their importance vary, of course, across species and environment. Note, for example, that cats can detect the difference in taste between sugar and saccharin, some snakes see via infrared light (heat), bats see via hearing (or echo-location), fish can smell water soluble molecules, dogs squirm at very high audio frequencies and detect cancerous scents that humans are quite oblivious to.

For humans at least, science has a reliable idea of the mechanisms involved in most senses. The taste of a molecule corresponds to which of the five (umami, sweet, sour, bitter and salty) receptors the molecule is able to activate (e.g., sodium glutamate, sucrose, acetic acid, quinine and sodium chloride respectively). Visual receptors allow us to see according to the wavelength of light that enters our eyes (red, green, blue) provided it is in the range 400–700 nm. Hearing uses mechanics within the ear to translate acoustic vibrations to sound: for example, frequencies of 16.35, 18.35 and 20.60 Hz correspond to musical notes C, D and E. Touch converts physical damage to sense receptors (heat, pressure) into sensory perception. Science knows the fundamentals: that glycerol makes things sweet, that blue and yellow can make green, that fundamental frequencies and integer related harmonics together sound nice, in ways identifiable by the chemistry and physics of the input information. Yet we do not completely understand the basic determinants (metrics) of how smell works at the odorant recognition level.

Smell is a process where small molecules meet large receptor proteins (factors of 1000's larger in size) and depending on the combination of David and Goliath, there is (or is not) a triggering of a signalling cascade that results in a smell perceived by the brain. But how do particular molecules cause (or inhibit) this process? It is not just in olfaction that the effect of one specific small molecule can cause a cascade of important processes. Other examples include the triggering of cells by hormones or the signal transmission in nerves by acetylcholine [1]. This combination of sensitivity (one molecule can initiate a complex chain of events) and selectivity (different molecules generate distinct perceived odours) is very remarkable [2]. Thus the question of how this works in principle extends beyond olfaction: what controls the very specific actions of neurotransmitters, hormones, pheromones, steroids, odorants and anaesthetics? How could the side effects of certain drugs be predicted? How do we control desirable and undesirable interactions of molecule and receptor? Answering questions like these would not only satisfy basic scientific curiosity, but might also provide a firmer foundation for drug design and development.

In important work that led to the award of the 2004 Nobel Prize in Physiology or Medicine, Axel and Buck isolated genes that coded for olfactory receptors, showing they belonged to the class of G-protein coupled receptors, GPCR [3]. Remarkable progress has been made over recent years regarding the genomics involved. However, whilst there is little doubt over what machinery is involved in the smelling (see Section 2), we still need to understand better the mechanics of how it does what it does. How can one understand the physics of the mechanisms that control the initial activation step when an odorous molecule meets one olfactory receptor? Though the crystal structure of soluble proteins can be determined, the detailed structure of olfactory receptors is still quite unclear because GPCRs are membrane proteins. Despite substantial progress [4,5] in producing large quantities of olfactory receptors (ORs), the ambitious aim of crystallizing these elusive proteins has yet to be achieved, thus there are still no detailed atomic structures of ORs. We note that whilst full structural information will surely be highly illuminating, a static picture of structure alone also may not tell us how odorant recognition is achieved.

### 1.2. What We Know and What We Do not Know about Odorant Recognition

As well as many of the biological mechanisms involved, we also know very precisely the molecular structure of most odorant molecules, and we can quantify a smell response. Response can be measured at the receptor level (the depolarization of the cell triggered by receptors) or by fluorescent magnetic resonance imaging (fmri) of the brain. It can also be measured by an individual's perception, though possibly less objectively. These parts of the puzzle, while understood, are difficult to manage because the number of degrees of freedom is so vast: the number of possible odorants may be in excess of 100,000 and the number of functional human receptor types is currently noted as 390 [6]. As a result, the number of different odorant-receptor combinations would be 390^100,000^ (*i.e*., practically unlimited). Many programs, for example E-DRAGON, calculate molecular descriptors based on the molecular structure of the odorants submitted [7]. However, cross-correlation between molecular descriptors and response patterns reveals that no particular metric, such as number of carbons or the presence of a particular functional group, represents truly faithful response patterns. A common metric for odorant description is usually the shape, possibly defined via a van der Waals space-filling model, that may be designed for particular odorants to fit within binding pockets within the receptor. There are certainly some correlations between the shape of a molecule and odorant receptor response, but likewise there are many cases where very different shapes produce the same pattern of activation of the odorant receptor repertoire because many ORs are broadly tuned. It has been suggested that Infrared (IR) vibrational spectra might be better predictors of smell than shape [8]. Programs like E-DRAGON, and those used for pharmaceutical design, do not directly implement vibrational spectra as an odorant metric. Furthermore, they explore the minimum energy (usually *in vacuo*) geometry of the odorant and do not account for effects at the binding site of the receptor or environment. Regarding typical IR spectra however, it is not certain that the correlation is any better than for shape for all known cases, though it does work for some [9].

One case where shape is clearly important in OR activation is the existence of odorant enantiomeric pairs that sometimes smell the same and sometimes smell different. The IR spectrum measured in achiral solution (the molecules are free to rotate) would not betray any difference between mirror-image related molecules, and yet the chiral environment of olfactory receptors would do. Therefore, IR absorbance without any geometrical consideration does not explain or predict odorant response; on the other hand, shape-based theories also do not explain why enantiomers sometimes smell the same and sometimes they do not.

Recent work [10,11] probes whether drosophila melanogaster identify chemical species on the basis of shape or not. In the experiments [10], four odorants were considered, along with their deuterated counterparts. The flies were trained to avoid one or other of the isotopic versions, and were then found that they can generalize their response to the other molecules based on which isotope of hydrogen was used. It can be concluded that the flies are responding to the presence or absence of deuterium, rather than molecular shape. The effect of shape on OR activation can be excluded in this case on two counts. First, replacing hydrogen by deuterium produces very little change in shape, yet the flies can distinguish the isotopes. Second, flies learn about deuterium from molecules with one shape, and then can generalize this knowledge to molecules of a different shape. One hypothesis is that the flies are responding to vibrational frequency of the C–H bond stretch: since deuterium has twice the mass of hydrogen, the frequency drops by about 40% following deuteration. This conjecture is given substantial support by a final experiment in which flies were allowed to respond to an odorant containing a nitrile (−C ≡ N) group, which has a vibrational frequency very similar to a C–D group. They reacted to it as they would to the deuterated odorants. This is important evidence suggesting that drosophila melanogaster can distinguish odorants by their molecular vibrations [10]. Of course, the drosophila olfactory receptors are of a different type to human receptors. However, the only commonality that is required to support the swipe card model is a hydrophobic receptor environment and an acceptable energy tuned gap (to the odorant vibrations). We address here how exactly these vibrations may be detected.

### 1.3. The Problem with Odorant Recognition

Humans can perceive by smell thousands of molecules, all small enough to be volatile, each of which activate a few olfactory receptors (it is very unusual that one odorant only activates one receptor). Further, humans can detect odorants at very small concentrations in air even 1 parts per trillion. The selectivity of these olfactory receptors is also especially remarkable considering that some odorants, may agonize or antagonize a receptor [12]. There are 390 functional olfactory receptors in humans [6] that can respond to 100,000 or more odorants, thus eliminating the concept of 1:1 receptor to odorant matching. Another reason that receptors cannot have evolved to identify individual molecules (at least not all of them) is their ability to respond to chemicals never encountered before. Thus olfactory receptors are versatile and accommodating and yet often discriminating and selective. Olfactory receptors are large, floppy transmembrane proteins, containing tens of thousands of atoms. Yet their abilities are rightly envied by scientists designing artificial noses and other sensors. How is this selective, sensitive, powerful, versatile, activation achieved?

Understanding the physics of odorant recognition at the receptor level means understanding how an odorous molecule (which we shall call M) initiates a measurable signal. There are potential analogies with vision, where a photon causes an initial molecular transformation [13]. In this paper, we seek to better understand the corresponding atomic-scale mechanisms by which olfactory receptors are activated by odorants. We shall develop the swipe-card paradigm for the selective activation of receptors by small molecules. This paradigm recognizes that the small molecule must have a shape that is, in some sense, good enough to engage with the receptor, but some other property or process is needed to yield a selective response. With recent evidence that vibrations may be indeed detected, we suggest that the likely property is a vibration in the odorant molecule. We attempt here to identify and, where possible, quantify the first signal transduction step in the receptor that results in the release of a G-protein. Processes at the glomeruli and olfactory bulb are beyond the scope of this work. Indubitably, there are important processes that control the overall perception [14] as the brain builds a scent perception from a number of receptors. Almost certainly any one molecule will be able to initiate a signal to the brain from a range of receptors. But the brain must have distinctive information to work with, and our concern here is just what molecular information determines whether a given receptor is activated and initiates a signal to the brain.

### 1.4. Competing Theories of Odorant Recognition

When odorant M reaches the hydrophobic cavity within the receptor, it will interact with amino acid residues of GPCR protein, which is comprised by seven transmembrane helices. Though considerable progress has been made regarding proton switch activation in rhodopsin [15] exactly how M activates the olfactory receptor is uncertain in detail. The orientation of M will fluctuate, influenced by weak bonds such as van der Waals and electrostatic interactions. These relatively weak interactions may stabilize an active configuration that might induce an on configuration of the receptor (which we term R) and M, which we may label R+M. This is the stage to invoke analogies with a key in a lock [16], or a hand in a glove [17] and also to recognize that not only must M have the right shape, but somehow the key must be turned. The lock-and-key principle requires that there is a good fit between two reactants in order to create the desired product, and it operates as follows. In the absence of the ligand, a receptor protein fluctuates about some average configuration, and with this is associated a free energy, *G_R_*. The average configuration is such as to minimize *G_R_*. Once a ligand binds to the receptor, there is a new average configuration for the receptor. The change in average structure can induce a signal, and thus corresponds to the key turning. This mechanism works very well for many receptors (see for example Sigala *et al*. [18]), and is likely to be the mechanism when a receptor is tuned to just one ligand (as presumably is the case for pheromones). Given the success of this mechanism in many known cases, it is natural to extend it to olfaction, as has indeed been done by Amoore in 1962 and added to by Moncrieff in 1967 (refs). However, for promiscuous olfactory receptors that are known to respond to multiple ligands, it is far from clear that this mechanism can explain all properties of olfaction. Indeed, systematic studies show shape alone is a poor criterion for predicting odour [19]. We thus look for complementary possibilities.

**Figure 1.**
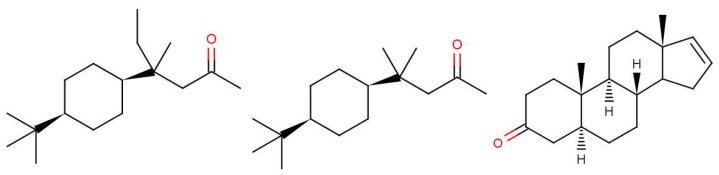
Contrast these three odorants: according to shape theory, which would you predict smell the same? From left to right; *cis* -ketone (4-(4-tert-butylcyclohexyl)-4-methylhexan-2-one), *cis-nor*-ketone (4-(4-tert-butylcyclohexyl)-4-methylpentan-2-one) and 5*α*-androst-16-en-3-one . *Cis* -ketone and 5*α*-androst-16-en-3-one have the same “penetrating urine odour” and *cis-nor*-ketone is practically/totally odourless [21].

In any theory of odorant recognition, the olfactant molecular shape must play a role, if only to let the scent molecule access key parts of the receptor. Theories of the initial actuation event fall into two broad categories. One class relies on olfactant molecular shape alone, a class that covers many structure-activity relation descriptions. Some level of fit is clearly necessary, but even a good fit is not sufficient (see Figure 1: the odorant must somehow activate the receptor. What turns the key in the lock? As we have seen above, one natural assumption is that the odorant causes a mechanical deformation of the receptor. To illustrate the problem, consider ferrocene and nickelocene. These molecules have different odours, and yet have similar shapes (for example see following figures). A systematic and extensive analysis of the problem by Charles Sell makes the point much more forcibly in our opinion [19].

A possible alternative model is what we have termed the swipe card picture [20]. It proposes that, whilst the shape must be good enough, other information characterising the odorant is also important. In lock-and-key models, a key of the right shape contains all the information to open the lock. In a swipe card (or keycard) model, the shape has to be good enough to fit the machine, but additional information is conveyed in a different way. Typical macroscopic swipe cards, like credit cards or hotel room cards, often encode the information magnetically. The specific swipe card model of odorant recognition we assess here uses a molecular vibration frequency as the additional information.

The theory of Turin proposes that an electron transfer occurs if the odorant has the right vibrational frequency: discrimination and activation are achieved by an inelastic electron tunnelling (IET) mechanism, dependent on the ability of the odorant to absorb the correct amount of energy. IET describes a phenomenon well known in inorganic systems. The swipe card description was conceived as a generalisation of models like Turin's original idea of conventional IET within a biological context, but we emphasise that there are important differences. In our own work [20] we made a critical assessment of Turin's basic ideas, showed that the ideas seemed robust, needing values of key parameters in line with those from other biological studies. Our present paper extends the analysis, generalizing the simpler model of the previous paper, and assessing possible physical realizations. In our earlier publication, we could find no physics-based objections to Turin's model of the signal transduction mechanism in odorant recognition, in which discrimination and activation are achieved by IET. An advantage of such models is that they are potentially predictive.

IET in inorganic systems is usually observed in circumstances that allow transmission over a continuum of energies. In biological systems, there are no equivalent continuous energy distributions. Our evaluation describing signalling times lets us compare the relative rates of non-discriminating tunnelling (characterized by an average time *τ*_0_; in this case, energy is given to some combination of host modes or other degrees of freedom) and of discriminating tunnelling (characterized by a time *τ*_1_). Successful selective activation requires the discriminating contribution (sensitive to the oscillator frequency *ω_o_* of odorant M) to dominate the non-discriminating contribution: *τ*_0_ ≫ *τ*_1_. We showed this to be the case in rather general and robust circumstances in our previous paper. We stress that our receptor models need to recognize three points:

1. First, there will surely be some shape constraints, though these may play only a small part in discrimination.
2. Secondly, there are dynamic factors (such as conformational change) that appear detectable by olfactory receptors, so a purely static model is not appropriate.
3. Thirdly, we need to consider both charged components of the receptor/olfactant system and also charge transfers during actuation.

Finally, we describe a quantized model for biological signal transduction at room temperature, a field of physics surrounded by controversy. Just as the initial events in photo-induced processes are very well described [13], a physically viable phonon-mediated mechanism is, we believe, well within the realms of reality as a putative signalling process.

Even though the main thrust of the paper concerns what happens when the olfactant encounters a receptor, it is important to recognize that this is just one—albeit a critical one—of the steps between there being an olfactant in the atmosphere and the brain perceiving some odour. The sequence of events leading up to odorant recognition provides a context and lets us estimate a timescale for the critical steps. Given this context, we assess the feasibility of this proposed biological spectroscope as an olfactory detector. This extends our previous discussion, with a closer look at just what the relevant biological components might be, and what would be reasonable values of the basic parameters. This allows us to identify some of the implications of the model, and especially what might prove significant tests. We also note that odorant shape and frequency are not always sufficient to define a smell, since other factors, such as conformational mobility, are certainly important for a large class of enantiomers. But shape and vibrational frequency go a long way towards defining odour.

### 1.5. Why Any Solution must Involve Physics

Turin's mechanism is a specifically quantum idea, partly because of tunnelling, but primarily because a quantum oscillator can only receive or give energy as quanta of specific energy. A classical oscillator can, of course, give or receive any amount of energy. Inelastic electron tunnelling is long known in the physical sciences [22–24] and in a biological context in reactions [25]; however, it is new to biological signalling, and has led to misunderstandings [26]. Turin's basic idea, leads directly to possible experimental and theoretical tests. We shall discuss his ideas critically, emphasizing the observations that confront these ideas most significantly. Our first main aim is to check any possible physics-based objections, in an extension of our earlier work [20]. Our second main aim is to see what existing experimental scent studies imply. Thus, we examine especially those molecules that appear to be problematic. Such problem molecules are, in fact, challenges to almost all theories of odorant recognition. It will become clear that vibration frequencies do appear important, but there are still limits to what can be understood in terms of odorant shape and vibrations.

In re-examining some of the ideas of how small molecules selectively activate receptors, we conjecture that the definition of signal generation here is repeated in other natural systems. Thus we have found a general rule that determines whether the two molecule types making up an enantiomer pair will smell the same or different [27], and this has implications for any model of signal transduction. We shall also discuss isotope effects [10], and the very striking observation that zinc nanoparticles available in the vicinity of the olfactory receptor can greatly enhance perceived odour intensity [28]. We note that often very subtle differences in molecular structure can drastically alter a scent, often in a surprising way, making scent prediction difficult. Evidence is emerging that, when shape information is combined with molecular vibrational data, good selectivity is possible [9]. For example, drosophila melanogaster can distinguish odorants by their molecular vibrations, and can even selectively avoid deuterated counterparts [10]. We note however, just as for shape, vibrations as a distinguishing characteristic alone is not enough. A swipe card model, going beyond the simpler lock and key ideas, can cover both requirements.

## 2. A Brief Summary of the Key Physical Processes Occurring During Odorant Recognition

### 2.1. Journey of the Odorant to the Receptor

Smell is a process where we directly interact with the world. Once the odorant is inhaled, it is only a short journey for this molecule (M) to interact directly with our central nervous system. The first stage on this journey takes M to the olfactory mucus, the 10–40 *μ*m thick covering of the olfactory epithelium [29]. The role of the olfactory mucus is not obvious, though it may simply moderate the concentration of odorants reaching the epithelium; it has even been suggested that the mucus serves as a separation column [30]. It has also been shown that diffusion of inhaled air towards the epithelium and its variable distribution inside the nasal cavity may be another way to differentiate scents before they hit the receptors [31]. The mucus layer contains odorant binding proteins (OBPs), small lipocalin carrier proteins whose role is unclear [32]. These OBPs have a high affinity for aldehydes and large fatty acids [33,34], so it seems likely their purpose is to assist transport of the largely hydrophobic odorants across this aqueous mucus layer to the epithelium. A further suggestion is that non-sensory respiratory cilia embedded in the nasal mucus aid odorant molecule transport. Also within this mucus layer reside biotransformation enzymes. The purpose of these enzymes is also as yet unclear. It is possible that they clear odorants away, or even metabolize them before they reach the receptor site [35]. It is usually assumed that the odorant is unaffected chemically on its journey to the receptor. However, comparisons of odours could well be affected even by small differences in metabolism, for instance from reaction rates depending on isotope, or chiral catalysts affecting enantiomers differently.

### 2.2. Recognition of the Odorant and Signal Initiation

Olfactory sensory neurons (OSN), traverse the epithelium and project cilia that extend into the mucus. Each OSN type projects cilia containing one particular type of olfactory receptor (OR); the number of ORs at the cilia varies according to species. The odorant M meets the OR by passing the mucus layer interface and docking at a binding site in the protein. The main thrust of our paper will be what happens when the odorant M reaches the olfactory receptor: what are the primary activation events that lead to a signal being initiated and a G-protein released, which is the primary action of a GPCR?

### 2.3. Signal Amplification and Processing

Once activated, the OR releases subunits of *G_olf_* in a series of local steps that are well understood [32,36,37]. The *G_olf_* activates the formation of adenyl cyclase III (AC), an enzyme which, in turn, activates an increase of second messenger cyclic adenosine monophosphate (cAMP). Then cAMP binds cAMP-activated cationic channels and cyclic nucleotide gated (CNG) signaling is released resulting in an ion channel opening and a *Ca*^2+^ and *Na*^2+^ influx. This results in a depolarization of the OSN, with perhaps subsequent amplification steps (possibly by as much as 85% [36]). The axons of the OSNs project through the cribriform plate to the olfactory bulb (OB). In the bulb, neural axons route to structures called glomeruli [3] which are discrete loci on the olfactory bulb. For each type of OR, the location to which they extend in the brain is the same in all subjects. One OSN expresses only one type of OR and there is a direct, non-branching route from OSN to glomeruli type which is referred to as zone-to-zone mapping [38]. The combinatorial pattern thus makes an impression on the brain which characterizes the smell of M. Functional magnetic resonance images can then reveal the regions of the brain activated by odorants [39]. There is evidence that the perception is at least partly a learned phenomenon [14]. However, this lies outside the scope of this paper, and is not discussed further. We make a clear distinction between the peception of smell and the depolarisation of a cell caused by OR activation: it is the latter that is our concern here.

## 3. The Olfactory Receptor as Vibrational Spectroscope

### 3.1. Shape, Weak Bonds and Vibrations All Matter

All the current theories acknowledge that the odorant must fit within the receptor, and must remain there long enough for a signal to be generated. Thus to some extent shape and the weak interactions between the odorant and the receptor must matter. However, as we saw above, this is not enough. Turin's assertion is that vibrational modes of the odorant matter as well [8]. As is elaborated below, the conjecture is that the receptor exploits inelastic electron tunnelling to detect the molecule's vibration frequency. We note that vibrational frequencies are of course strongly dependent on the odorant geometry.

### 3.2. Inelastic Electron Tunnelling: Turin's Model

**Figure 2.**
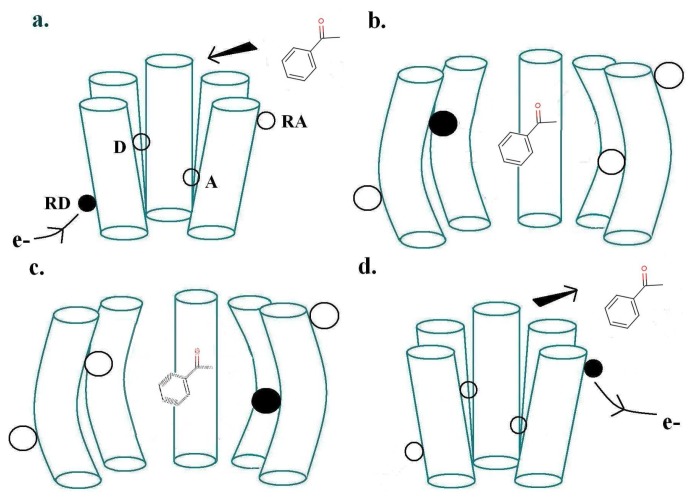
A scheme for the proposal of electron transfer in the olfactory receptor. Only 5 transmembrane helices (of the 7 in total) for the olfactory receptor are shown (cylinders) here for clarity. (**a**) The odorant approaches the receptor, meanwhile an electron moves to position RD on a helix; (**b**) The odorant docks at the ligand binding domain, the overall configuration of receptor and odorant changes, meanwhile the electron tunnels within the protein to D and it spends some time there; (**c**) The electron jumps from D to A causing the odorant to vibrate; (**d**) The odorant is expelled from the ligand binding domain and the electron tunnels within the protein to site RA. Signal transduction is initiated with the G-protein release.

The physics of Turin's mechanism was assessed previously [20], finding it consistent with other biophysical mechanisms. Here our aim is to confront theory with empirical observation, a more important test. Turin's model envisages two special points where the receptor and odorant M make contact: a donor D linked to a source of electrons, and an acceptor A linked to an electron sink. The electron transfer event from donor D to acceptor A—because charge is moved—will change the forces on M. This sudden change in force causes M to change vibrational state. Energy must be conserved overall. If the transfer is to occur, as an inelastic tunnelling event, the electronic energy difference between D and A sites must match the vibrational energy taken up by M. The transfer of the electron to A triggers a conformational change of the receptor, which produces the release of the *α*-subunit of a neighbouring G-protein (G), which via subsequent processes outlined above, initiates the large influx of *Ca*^2+^ ions into the cell, thus initiating a signal communicated by consequent firing of neurons to the brain. Figure 2 shows these first events at the ligand binding domain (LBD). The process just described, inelastic electron tunnelling, is very well established in inorganic systems. Its commonest form has tunnelling between metal junctions bridged by a single molecule [22] in an insulating gap. The molecule within the gap can be identified, and even its orientation revealed. But metal electrodes have a continuum of energies, so it is hard to resolve the weak inelastic transition superimposed on the dominant elastic transition to this continuum of states, even at the very low temperatures usually used. This problem is avoided in the system hypothesized for odorant recognition. In the olfactory receptor, the assumption is that D and A have discrete energies, and—to an extent that can be calculated [20] (see below)—there is essentially no elastic transition, and competition comes from weak transitions where energy is taken up solely by host vibrations. This is important: extracting a weak inelastic adjunct from a larger non-discriminating signal could be an unnecessarily noisy job for the brain, especially at ambient temperatures. With discrete initial and final states, the inelastic transition can be far better resolved. Of course, charge transport in the nose to D and from A must be inherently different in some ways, but this does not seem to raise any insuperable problems. For example, conducting polymer nanotubes (CPNTs) conjugated with human olfactory receptors have recently been created [40]which act as field-effect transistors (FETs). These biosensors are sensitive to a current increase upon odorant binding, even at low concentrations.

### 3.3. How the Charge Moves: Inter-Chain or Intra-Chain Charge Transfer

**Figure 3.**
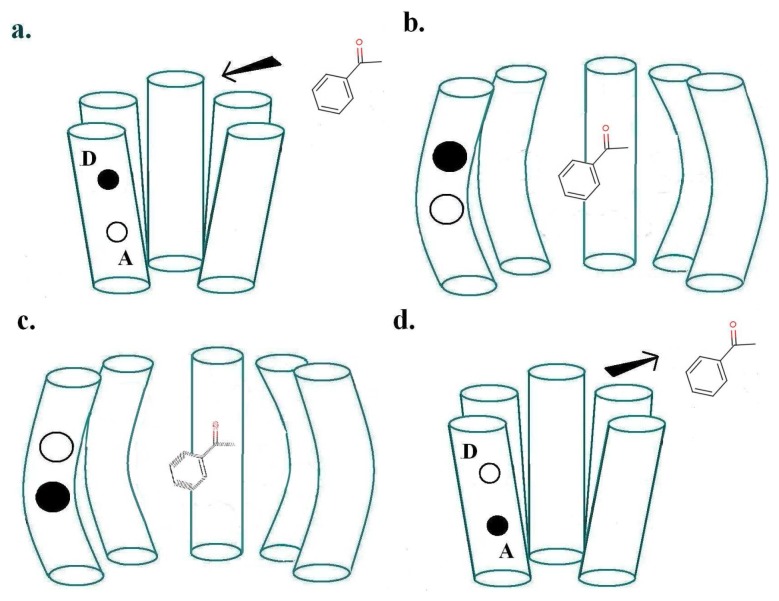
A scheme for the proposal of electron transfer in the olfactory receptor with *intra*-protein electron transfer. Only 5 transmembrane helices for the olfactory receptor are shown (cylinders) here for clarity. (**a**) The odorant approaches the receptor, meanwhile an electron is present at donor site D; (**b**) The odorant docks at the ligand binding domain, the overall configuration of receptor and odorant changes (**c**) The electron jumps from D to A, causing the odorant to vibrate (**d**) The odorant is expelled from the ligand binding domain.

For a biological inelastic tunnelling process, two optimal routes could be proposed for the moving charge via the receptor helices. These are depicted in Figures 2 and 3.

As usually described, the inelastic tunnelling transition takes the electron from donor D on one of the olfactory receptor's polypeptide chains through the odorant to an acceptor A on another polypeptide chain. This *inter*-strand picture (see Figure 2) was used in our earlier analysis. But it is not necessary that the electron passes through the molecule, the word “through” meaning that the odorant wavefunction is a significant part of the transition matrix element. A sudden change in electric field at the odorant is sufficient to cause it to change vibrational state. So an *intra*-strand charge transfer transition (see Figure 3) is a satisfactory alternative.

We can show by explicit calculation (see Appendix A for simple analytical examples; more detailed results will be published separately) that the couplings between the odorant vibrations and the electron transition can easily be of the same size for both *inter*- and *intra*-chain charge transfers. Perhaps the main advantage of *intra*-chain charge transfer is that there is no need for any long-range motion of charge to re-set the donor and acceptor to their original states. It is possible, for example, that the original *intra*-chain electronic states will be recovered simply by the olfactant leaving the receptor, but that is pure speculation.

In the case of *inter*-chain charge transfer, one might assume that this single electron current is what starts the next stage in the series of local processes mentioned in Section 2.3. In the case of *intra*-chain charge transfer, there may be no current in the same sense, but there will be a significant, possibly short-lived, electrical dipole moment. One conjecture might be that the electric field from this transient dipole initiates the next stage.

### 3.4. Time Scales Involved

Experimentally, olfaction occurs over milliseconds, decidedly slowly when compared with most processes at the molecular scale (see Table 1). In the model of Figure 2, the likely rate-determining steps involve transport of an electron to D or removal of an electron from A. For the receptor to operate it requires an electron in the donor that is free to make the transition to the acceptor. Producing this initial state requires some input of energy, though this is not expected to be problematic as voltages of order 0.5V are certainly available in cells. The precise mechanisms are not known, but we can make some simple rough estimates for different options. First (Model 1), suppose that charge *q* must diffuse a typical distance *L* with diffusion constant *D* (related to the mobility *μ* = *qD*/*kT* by the Nernst-Einstein relation). Assuming there is no driving force (bias) the characteristic time will be 1/*τ_X_*_1_ ∼ *D*/*L*^2^. Secondly (Model 2) suppose the charge motion is diffusive, but biased by a field *U*/*L*; here *U* may be an electrochemical potential. The drift velocity is *μE* = *μU*/*L* and distance to move is *L*, so 1/*τ_X_*_2_ ∼ (*qD*/*kT*)*U*/*L*^2^. With q as one electronic charge, and *T* as 300K, the biased motion is faster for *U* bigger than around 1/30 volts. With *D* ∼ 10^−4^*cm*^2^/*s*, typical of liquids, and *L* ∼ 100 nm, one finds *D*/*L*^2^ ∼ 10^6^sec^−1^. *U* may well be larger, say 0.5 V. Whilst these arguments are speculative, it is not unreasonable to expect characteristic times associated with charge transport to be of the order of a microsecond. The Table shows that, for the model of Figure 2, electron transport to the donor or from the acceptor could be relatively long, assuming incoherent hopping, transfer, of the electron at rates typical of other biological systems. No matter how fast the tunnelling event, the re-population of D (D must be replenished systematically) and re-emptying of A puts a bound on how many tunnelling events can occur in one receptor. In the *intra*-chain charge transfer, Figure 3, it is simply necessary for the charge transfer to be reversed, *i.e.*, for the electron to return from A to D. This should not need transport over any extended distance, but may need the olfactant to leave the receptor.

It is tempting to assume that only one electron can pass from the time the odorant enters the receptor until it leaves. However, we have no evidence on this point. It is certainly possible to devise models in which more than one electron would pass.

### 3.5. Which Electronic States are Important?

So far, we have simply observed that as an electron transfers from D to A, it alters forces on M, so causing a change in the odorant's vibrational state. Such behaviour has parallels in many other solid state systems. Until more is certain about the receptor structure, doubts must remain as to precisely which groups D and A correspond. We return to this question in Section 5.1. We have assumed that D and A are relatively localized, and that the odorant molecule M in the receptor is close to either D or A or, perhaps more probably, to both of them. The donor and acceptor species will have discrete energies, unlike the electrodes in most inorganic inelastic tunnelling experiments.

Two distinct types of transition can be identified immediately. In one, there is a direct transition from D to A that is modulated by the presence of the odorant, and this was the case considered in [20]. In the second type, there is an electron transition onto the odorant, followed by an incoherent second transfer to A. This second category, with electron transfer into the odorant molecule M (*intra*-molecular tunneling [41,42]) requires available molecular orbitals of M close in energy to those of the donor and acceptor. Typically this requires re-hybridization between the adsorbed molecule and receptor to make the energy differences suitably small [41,42]. We can rule out these ideas for OR activation, ultimately because the HOMO-LUMO (highest occupied and lowest occupied molecular orbitals, respectively) gaps of odorants are typically large, of order 10 eV.

Thus we examined the case of *extra-*molecular tunnelling [20], electron transfer near the odorant molecule, which seems more probable. We shall discuss the possible natures of D and A later, but, for the moment assume that D and A can be either occupied by an electron or unoccupied by an electron, and that the states (D occupied, A unoccupied) and (D unoccupied, A occupied) differ in energy by 0.1–0.2 eV. The odorant M may facilitate the transition (see Appendix B) but there is no need to assume a quasi-stationary state in which M hosts the electron for any length of time.

We may consider here two possibilities for the electron tunnelling path: (1) the electron crosses between opposing helices (*inter*-helix crossing), possibly passing through M, which will affect the relevant tunnelling matrix element; (2) the electron moves within one helix (*intra*-helix crossing). The original model [20] assumed *inter*-helix crossing; there is little difference in the physics between the two cases, see above, but they have different implications in determining the chemical natures of molecular units D and A, and just how they form part of the receptor structure.

As noted, we regard D and A as localized, with their relevant electronic states compact compared with the distance between them. Sensible guesses from the distances between helices suggest a typical value of around 8Å. This would be realistic if we believe the likelihood that these electron source/sinks are amino acids and if we compare to distances between important residues for rhodopsin [43]. Site-directed mutagenesis [44,45] studies have determined that for odorant recognition in MOR-EG there are nine amino acids involved directly at the binding site, with Ser113 being a crucial H-bond donor for odorants with aliphatic alcohols. It is noteworthy that none of the nine is strongly conserved (see Figure 4 of [44]), and indeed some are at sites that are highly variable. Thus they can only be associated with binding or modifying the donor and acceptor characteristics; Katada *et al.* [44] associate them with binding. For electron transfer via the odorant, all that is needed is a combination of firstly the right quantized vibration frequencies and secondly a rapid electronic charge transfer. In the tunnelling transition between these two states, what the impulse is that drives vibrational excitation becomes the key question.

### 3.6. Odorant Vibrations

To activate the receptor the odorant frequency has to stand out against the many background host modes, such as the C-H stretch vibrations that are abundant in the environment and occur at around 0.36eV (2911.3 cm^−1^) [46]. The couplings will depend on an effective charge and on the root mean square amplitude of vibration. We can estimate a root mean square displacement (rms) thermal atomic displacement of these stretches using:

(1) $${\bar{x}}^{2}=\frac{1}{2}\frac{\hbar }{M\omega }\coth\left(\frac{\hbar \omega }{2k_{B}T}\right)$$

which with *M* = 1 for hydrogen in atomic units, and *ћω* = 360*meV*, we see a root mean square amplitude of 0.076Å. This is small, which is reassuring. Estimates of effective charges in the receptor environment are not simple. A potential problem is the abundance of C-H modes, even if each individually were weakly coupled. Turin [8] has suggested that frequencies around common CH stretches are blind-spots and the appropriate receptor type does not occur.

### 3.7. Odorant Vibration Coupling

The Huang-Rhys factor S, is a measure of the coupling of the tunnelling transition to the vibrational mode of the olfactant M. To be more precise, it is a measure of the change in force experienced by the vibrational modes following the transfer of an electron from the donor to the acceptor site. It can be calculated using readily available electronic structure codes, and this can be done accurately for free odorant molecules. The same methods give vibrational mode frequencies. The Huang-Rhys factor thus allows us to make predictions for different odorants, based on the strength of this factor. The predicted values (to be discussed in a separate publication) do indeed lie in the useful range 0.05-0.3. The couplings are thus strong enough to be detectable by inelastic tunnelling, but not so strong as to have 2 or 3 phonon processes that obscure discrimination.

### 3.8. Key Non-Radiative Transition Probabilities

**Figure 4.**
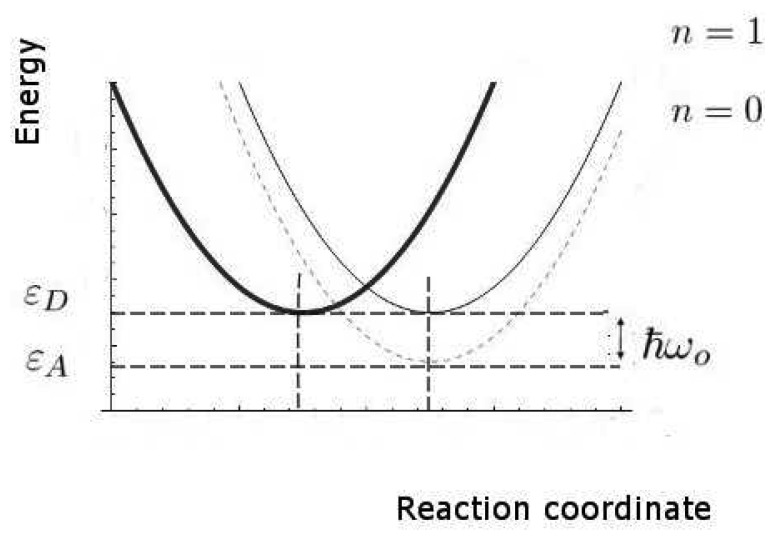
A configuration coordinate diagram to show the initial state (the left curve) and the final state (the right curves) where there are two options: the inelastic (*n* = 1) *versus* the elastic (*n* = 0) route.

We now estimate the key rates and probabilities for the inelastic tunnelling events. We shall need to calculate the relative rates of discriminating and non-discriminating tunnelling, since this determines whether a signal might be initiated that would allow the brain to distinguish odours. These rates are characterized by the two timescales, *τ_T_*_0_ and *τ_T_*_1_, for transitions without and with excitation of the olfactant vibration respectively, see Figure 4. Secondly, we need to estimate the spectral resolution that might be achieved. Thirdly, we need to make some estimate of limits, even crude, of absolute rates (effectively, absolute values of *τ_T_*_0_ and *τ_T_*_1_), so that we can verify that the timescales can be met.

In these calculations, we shall make use of the large body of standard non-radiative transition theory. This theory takes various forms, including Huang-Rhys theory and Marcus theory, having many elements in common, but differing because of the specific cases at which they were first aimed. The standard elements of these theories include the idea of a configuration coordinate (reaction coordinate, see Figure 4), and the assumption of processes sufficiently slow that the usual perturbation approaches to transition probabilities (like the Fermi Golden Rule) can be used.

We remark that, even in classical physics, the charge transfer from D to A would cause a change in force that would, in turn, change the vibrational state of the molecular oscillator. A quantum description makes two relatively simple changes. First, there is a more complete description of the charge transfer event, here a coherent event in which the electron loses energy and the molecule gains the same amount of vibrational energy. Secondly, the vibrational energy of the molecule can only change by discrete amounts, the vibrational quanta corresponding to their characteristic frequencies. In general, an odorant will have multiple vibrational modes but, for simplicity, we concentrate on the one mode presumed dominant in olfaction. At ambient temperatures, that mode will normally be in its ground state before excitation. For a typical odorant vibrational quantum of *ћω*_0_ = 0.2eV the probability *P_ex_* of that mode being in an excited state $P_{\text{ex}}=\text{exp}\left(-\frac{\hbar \omega_{0}}{k_{B}T}\right)$ is very small indeed, about 0.0006, for normal human body temperature, *k_B_T* = 0.027eV. An interesting case that previously alleged to prove vibration based theories wrong becomes poignant here: two isomers, methyl cyanide (*CH*_3_ − *C* ≡ *N*) and methyl isocyanide (*CH*_3_ − *N* =̇ *C*), despite having very similar higher frequency vibrational spectra, smell quite different. Wright's objection then was that “for quantum reasons, the vibrations in question [for smell] must have a rather low vibrational frequency and probably lie in the range 500 to 50 wave numbers” [47], and differences in the lower frequency region of the spectra explained the discrepancy in smell. We note here that, for this suggested refutation, the IR spectra do indeed differ [48], most notably in the 2,200 cm^−1^ region where methyl isocyanide exhibits much stronger IR absorbance, and so fits comfortably within predictions described by the model used here: it is not a refutation of a vibrations-based model.

The electron transfer between the initial state on D and the final state on A couples to the vibrations of the molecule M and of its environment. In effect, forces on the atoms change because charge has moved. The receptor environment will surely have some effect on the details of the vibrational modes of the molecule M, but we shall assume that the high frequency modes associated with selectivity are not altered greatly from those of the free molecule; this assumption could be removed in larger calculations. The environment, including the “soft” floppy protein backbone fluctuations observed in protein dynamics, we take to be a collection of low frequency oscillators that couple only very weakly to the mobile charge. This is a less accurate approximation for the amino acids near the donor and acceptor sites than for the remote regions [49]. This will be able to be approved once we are confident of the location of the donor and acceptor sites, and have reliable geometries for the receptors. We can then obtain the electron transfer rate, as in [20], using the standard theories of non-radiative transitions based on Fermi's golden rule [20,50–52]. The final expression involves an electronic matrix element (Appendix B) and factors that describe how readily the molecule and environment oscillators can take up energy. These factors are conveniently expressed in terms of a dimensionless Huang–Rhys factor *S* (the molecular relaxation energy for a mode divided by its vibrational energy quantum) for the molecular modes, and a reorganization energy for the environment modes.

For inelastic tunnelling to be effective, the Huang–Rhys factor *S* for the molecule should lie roughly in the range 0.01 to 0.3. For *S* <0.01 inelastic events will probably be too rare. For *S* >0.3, multiphonon events will begin to be a problem. The environmental reorganization energy should also be small, so that the electron transfer is unlikely to be achieved using the softer environmental modes alone. It is the coupling to these environment modes that limits the spectral resolution of the inelastic tunnelling mechanism through processes (for example) where the vibrational energy needed, *ћω*_0_ is the sum of a molecular mode energy *ћω_M_* and an environmental mode energy *ћω_e_*. In at least some systems (Marcus, private communication) this environmental reorganization energy is very small, but calculations of a full molecule plus receptor system are desirable.

### 3.9. The Influence of the Environment

The environment of the odorant varies from air to wet mucus to a dry hydrophobic region. At the point of interest within the binding domain between the membrane which is surrounded by hydrophobic phosphorolipids the odorant is in a very dry environment. That is, vibrations around the odorant are typically not moving charges (the important moving charges are only the atoms on the odorant and the itinerant electron). The inelastic tunnelling model is very sensitive to the reorganization energy of the environment, the host vibration couplings: *λ*. With the parameters chosen above, for values of *λ* below about 30 meV, the inelastic channel dominates, but increasing *λ* to above 62 meV would mean that the olfactant environment plays a dominant role. In terms of Marcus theory, we must consider contributions to the reorganization energy from both inner shell regions (*λ_i_*) and outer shell regions (*λ_o_*) [25].

For the inner shell, we assume harmonic modes, and have a contribution:

(2) $$\lambda_{i}=\frac{1}{2}\sum_{\alpha }k_{\alpha }Q_{\alpha }^{2}$$

where *α* runs over modes. As indicated above, most of these modes are soft, with low energies. There will be some modes with higher energies, like CH vibrations, but all may be weakly coupled. For the outer shell element *λ_o_* it is usual to use a continuum picture, estimated from the polarizability of the environment:

(3) $$\lambda_{o}=\frac{{\left(\Delta e\right)}^{2}}{4\pi {ɛ}_{o}}\left(\frac{1}{2r_{1}}+\frac{1}{2r_{2}}+\frac{1}{r_{12}}\right)\left(\frac{1}{D_{\text{op}}}-\frac{1}{D_{S}}\right)$$

where *r*_1_ and *r*_2_ are characteristic radii, *D_op_* is the square of the refractive index (the fast response dielectric constant) and *D_S_* the static dielectric constant (the slow response dielectric constant), and Δ*e* is the charge that is transferred. In essentially non-polar environments, such as the hydrophobic ligand binding domain, the charge transfer has little effect, and the outer shell nuclei move very little. There is good reason to assume that these reorganization energies for the olfactory system will be small (see also sections below). We note that the photosynthetic bacteria *Rhodobacter capsulatus* which has values of *λ* below 30 meV at room temperature. These possible environmental vibrational excitations could be calculated once a detailed structure for the receptor system is known.

### 3.10. The Electronic Transition Matrix Element |t|

The electronic matrix element *t* for a non-radiative transition is never trivial to calculate accurately. This is especially true when overlaps are small, as here, when the electronic states of D and A have very small overlap. Quite possibly *t* would be negligible in the absence of the olfactant. Certainly *t* will be different when the odorant is present, partly from changes in geometry, but also because of extra terms in the wavefunction (cf. Appendix B). For a rough estimate, we might consider single molecular orbitals, giving an effective hopping energy of: *t* = *υ*^2^/(*ε_M_* − *ε_A_*), where *ε_M_* is the energy level of the relevant odorant orbital and *ε_A_* of the acceptor, taking the appropriate highest occupied (HOMO) or lowest unoccupied (LUMO) molecular orbitals. For most olfactants, the HOMO and LUMO energy difference can be as big as 10eV. The hopping integral *υ* will not usually exceed 0.1 eV, determined from the strength of hydrogen bonds between the donor, acceptor and molecule. Better knowledge of the atomic structures of likely D and A units would allow better estimates of this parameter. Thus, for instance, Newton *et al.* calculate the matrix element in *Fe*^2+^ − *Fe*^3+^, from the overlap of the orbitals from these two iron atoms [53]. However, we cannot usefully attempt such a calculation without better indications of what the important groups and their orientations. Again, we can compare our system with experimental data for *C. vinosum*, from which, when the experimental parameters are inserted into a similar equation to 4 (with the same assumptions of non-adiabacity and low temperatures), then the matrix element obtained is 2.4 meV. This suggests our estimates have the right order of magnitude [53]. Fortunately, the proportions of transitions with and without olfactant vibrational excitation is essentially independent of *t*.

### 3.11. Discriminating between Molecules

The rate equation for tunnelling with or without olfactant vibrational excitation [20], can be summarized as:

(4) $$\frac{1}{\tau_{D,0\to A,n}}=\frac{2\pi }{\hbar }{\left|t\right|}^{2}\frac{\sigma_{n}}{\sqrt{4\pi k_{B}T\lambda }}\text{exp}\left(-\frac{{\left({ɛ}_{n}-\lambda \right)}^{2}}{4k_{B}T\lambda }\right)$$

where *n* = 0 for the non-discriminating channel when the olfactant is not excited (or the wrong olfactant is blocking the electron route) and all energy is taken up by host vibrations. For the discriminating channel, where the olfactant takes up this energy, *n* = 1. Typical values of the important parameters, given in the Table 2, indicate *τ_T_*_0_ ∼ 87*ns* and *τ_T_*_1_ ∼ 0.15*ns*. These satisfy the condition that *τ_T_*_1_ ≪ *τ_T_*_0_ by a substantial margin. The discriminating inelastic channel dominates the tunnelling between these states with discrete energies. This is, of course, the opposite of what is found for inelastic tunnelling involving metal electrodes with their continua of initial or final electronic states.

**Figure 5.**
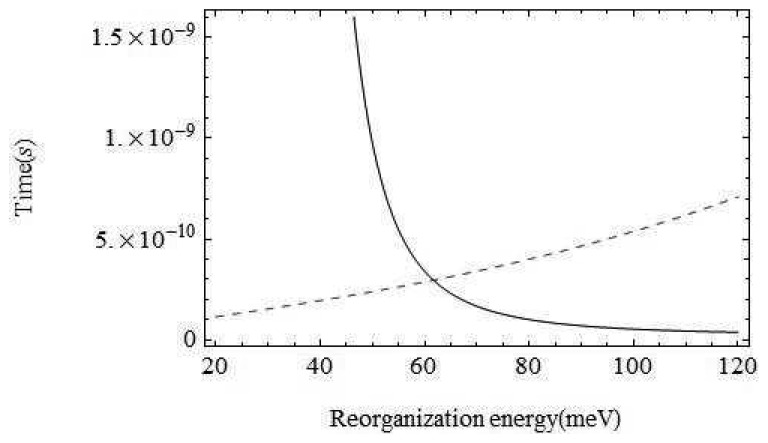
A plot to show the time (s) for an inelastic transmission (red, thin line) versus the elastic transmission (pink, thick line) all parameters given in the table are constant, the variable is the reorganization energy *λ*.

Perhaps the least certain parameter is the reorganization energy, *λ*, associated with the environment. If the environment were strongly coupled, the environment modes could take up most of the electronic energy and discrimination based on the olfactant mode would be ineffective. In Figure 5 we show a plot of the characteristic times for the channels with and without olfactant vibrational excitation as a function of *λ*. The figure shows that tunnelling primarily mediated by environment modes would dominate for values of *λ* > 62*meV*. Weak coupling to the host modes is crucial. Without a very detailed receptor structure, it is hard to check this further, but there are systems for which reorganization energies are in an acceptable range (Marcus, private communication).

## 4. Challenging Cases

The previous sections examined the basic physics of inelastic electron tunnelling as a potential critical step in olfaction. Even though the underlying physics appears viable, with credible parameters [20], the ultimate test is experiment. So how well do these ideas fit the observed phenomena of olfaction? We have chosen a set of examples that might challenge the role of inelastic electron tunnelling. From these, shown in Tables 3–5, we analyze and address implications. In several cases, further experiments are suggested.

### 4.1. Isotopes

**Figure 6.**
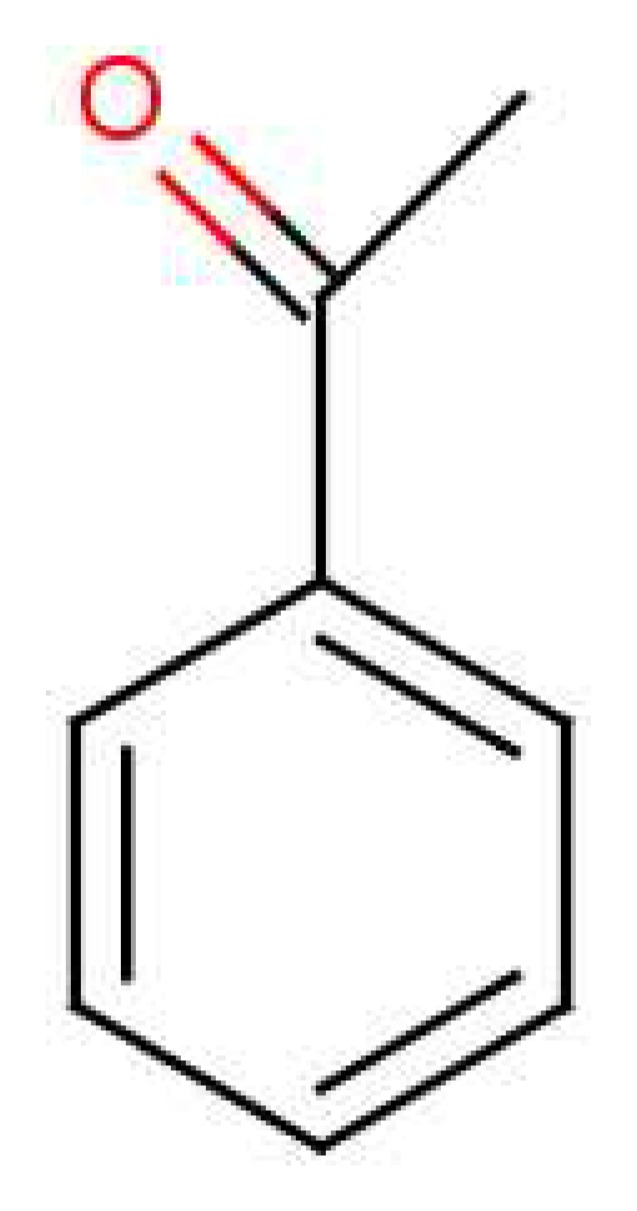
Acetophenone and acetophenone-d8.

Any isotope (see Figure 6) dependence of scent is inconsistent with standard notions of discrimination due to shape. However, humans and drosophila can indeed discriminate between isotopes in some cases, and drosophila can be trained to respond in a way that illustrates this [10]. Certainly there have been experiments that gave no evidence for an isotope effect [63], and the effect is relatively subtle. But the picture emerging leaves little doubt that there is an isotope effect [64]. It is possible to invoke special effects, such as isotope-dependent chemical reactions en route to the receptor, but the obvious effect of the isotopic mass difference is on the vibrational spectrum.

Isotope dependence gives an opportunity to measure the sensitivity to the energy separation of D and A for those responsive receptors, since the model parameters *t* and *S* remain essentially the same. We can predict the isotopic change in vibrational frequency Δ*δ* and see how that relates to observed discrimination and non-discrimination. Density Functional Theory (DFT) computations using a B3LYP functional and the basis set 6-311+G(d,p) for acetophenone show that the largest shift in the IR spectra occurs towards the higher frequency end. For a simple oscillator in which only the proton moves, the frequency for a deuteron would be smaller by a factor of $1-1/\sqrt{2}\approx 0.29$, or about 800 cm^−1^ for these modes. Assuming this cluster of modes is significant in olfaction, the shift Δ*δ* ∼ 800*cm*^−1^ should readily suffice to disengage activation for at least one receptor. Possibly even a much smaller shift would suffice. Such psychophysical tests on humans and behaviour studies on drosophila can be strengthened with discrimination at the glomerular level (as opposed to the perception level, which is sometimes contentious [58]) by using calcium imaging [59]. This class of experiment could definitively establish discrimination and would be a desirable next step to establish the phenomenon of isotope discrimination at the receptor level free from ambiguity.

### 4.2. Structurally Similar Odorants with Different Thresholds

**Figure 7.**
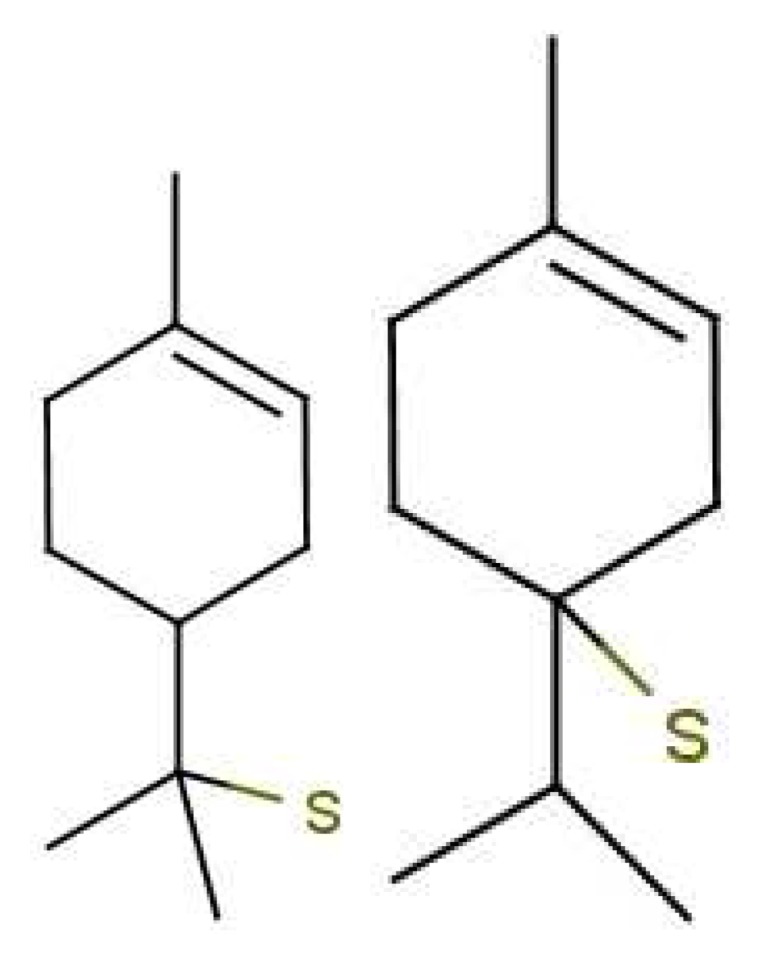
Sulphur compounds p-menthene-1-en-8-thiol and a stereoisomer. The latter smells 100,000 times weaker [55].

Examples where similar odorants have differing thresholds highlight the difference between affinity and efficacy. In olfaction, we define the propensity with which the odorant populates a receptor as affinity (typically binding), and the propensity with which it activates the receptor once there as efficacy (typically actuation), though the distinction is not as clear-cut as it might seem [65]. Affinity may include the rate of diffusion to particular receptor sites, the ability for it to cross into the hydrophobic domain, and the ability to make necessary contacts once there and “bind”. Affinity may also include how long the odorant remains within the ligand binding domain, and this time may impact on efficacy. One possible explanation for the differences observed in Figure 7 is given by Zarzo [66] where it is realized the presence of P-450 enzymes may cause differing interactions with odorants and hence altered responses at the affinity level. Although efficacy and affinity cannot be treated as entirely independent, one can make a cautious separation of the two.

Odorants that have very similar structures will usually have similar affinity for a given receptor site. However, there can still be differences in efficacy within our swipe card model. These could come from differences in *t* and/or *S*, and so might underlie the differences in perceived odour. For example, one receptor tuned to detect the SH stretch will receive quite different signals when the S-H axis is differently oriented within the receptor. Odorants with very similar affinities can provide us with good examples to measure Huang-Rhys factors *S* and compare these with observed efficacy, to see if actuation may be accurately described by our simple formulae.

### 4.3. Antagonists

**Figure 8.**
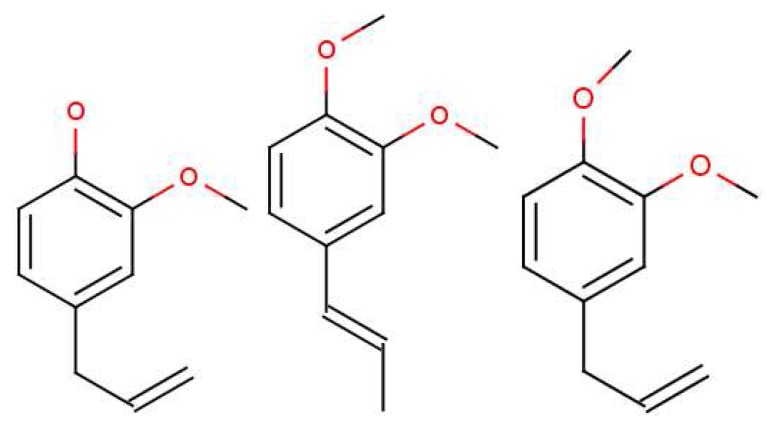
Eugenol (EG) (left) is an agonist of olfactory receptor MOR-EG, methyl-isoeugenol (MIEG) (middle) is an antagonist and methyl-eugenol (MEG) (right) is an agonist.

Oka *et al.* [56] state emphatically that their own results, and those in previous reports, clearly demonstrate that “antagonists tend to be structurally related to the agonists, as is often the case for other GPCRs”. This conclusion may not be consistent with shape-based theory; the receptors would have to be incredibly sensitive to always respond in different ways to very similar ligands (there would have to be as many receptor types as there are smellable molecules) but could be consistent with a swipe-card model, including cases where odorants can be differentiated spectrally. Comparing eugenol (EG) and methyl-isoegenol (MIEG) in Figure 8, we might surmise that it is the difference between the OH and O−CH_3_ modes that accounts for the difference in their perceived odours. However, examining the whole set from this study reveals that other mOR-EG receptor agonists, such as methyl-eugenol (MEG), do not possess the OH stretch either, though perhaps they do have a more suitable shape to fit the receptor site and D/A contacts. These results strongly suggest that what is needed is a combination of suitable parameters, *δ*, *t* and *S*. Both the fit and the correct vibrations are necessary. Oka *et al.* [56] have provided a set of odorants with varying levels of antagonism (and perhaps thus efficacy). Again, a good model for *S* may be able to account for any trends in *Ca*^2+^ response.

Antagonists remain puzzling in some cases. For example, Drosophila avoids CO_2_ whilst suppressing this aversion when it is associated with food sources, in which case odorants present in such foods directly inhibit CO_2_-sensitive neurons in the antenna. Some such odorants have been identified [67]. These antagonists include 2,3-butanedione (CH_3_CO)_2_, butanal (CHO)(CH_2_)_2_(CH_3_), pentanal (CHO)(CH_2_)_3_(CH_3_), and hexanol (COH)(CH_2_)_5_(CH_3_). They do not resemble CO_2_ in shape, and hexanol lacks a C=O unit. As regards vibrations, one interesting, if puzzling, feature is that all molecules have a vibration with frequency close to that of the infrared inactive symmetric stretch of CO_2_.

### 4.4. Smell the Same but Have Different Structures

**Figure 9.**
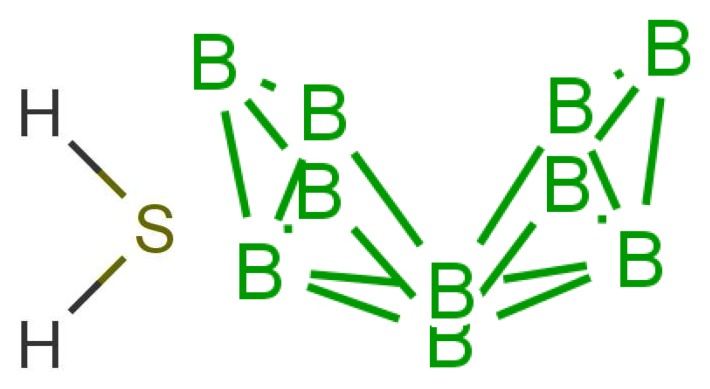
Hydrogen sulphide (left) and decaborane (right). Both smell sulphurous.

Similarity of smell suggests that both molecules shown in Figure 9 activate at least one receptor in common. The commonality in the cases shown may be that they share similar vibrations at around 2,600 cm^−1^. Thus assuming, a receptor with tuning *δ* corresponds to sulphuraceousness, we can use this example as a model for sufficient combination of *δ*, *t* and *S* in decaborane, although it is not the endogenous ligand (we assume that hydrogen sulphide is). Calculations using the B3LYP functional and the 6–31G** basis set indicate a difference between the H_2_S symmetric stretch and the strongest IR absorbing BH stretch in decaborane (around the 2,600 cm^−1^ region) be around 25 cm^−1^ (this result verges on the limits of accuracy for small energy difference calculations in DFT). We nonetheless surmise that the sulphur receptor must have a range of detection at least this amount, and so could be tuned to 2,600 ± 25 cm^−1^ for example. Modelling the “perfect” sulphuraceous receptor we could predict when some odorants do or do not smell sulphurous, particularly in examples which are surprising (*p*-menthene-1-en-8-thiol smells of grapefruit predominantly, see Figure 7) by again calculating *S.* Determination of a human olfactory receptor responsible for “sulphur” detection would provide a good starting model to compare and contrast this example and the sulphur containing examples in Figure 7.

### 4.5. Smell Different with the Same Structures

**Figure 10.**
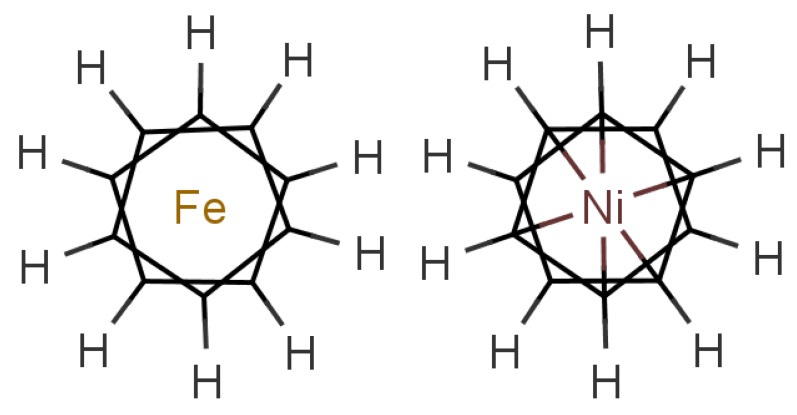
Ferrocene (left) smells “spicy” versus nickelocene (right) smells “oily-chemical”.

In Figure 10 are shown some striking exceptions to shape theories. Ferrocene and nickelocene smell different, yet they appear to have similar shapes and probably similar tunnelling matrix elements. Presumably, one molecule fails to activate at least one receptor that the other activates. Their different vibrational spectra might explain the difference, and this can be estimated (see Appendix C). This should guide us to what difference renders one odorant undetectable. We know from other systems (e.g., hydrogen sulphide and decaborane) that a difference of less than ∼25 cm^−1^ is unlikely to be detected, so presumably there must be a larger difference between the vibrational quanta of the key modes for ferrocene and nickelocene.

Cases where the structures are the same raise some broader issues. In the present case, the similarities of shape and interactions of ferrocene and nickelocene mean that they are likely to spend similar times in any receptor. Even when one has the wrong frequency, there could also be a weak contribution from tunnelling made possible by environment modes alone. Will there still be a different signal for that one receptor that will impact on the perceived scent? The point is that we have a series of steps: an electron must become available in D, a tunnelling transition must occur, and the electron must be removed from A. If the electron transfers into D or from A are slower than the tunnelling transition with only environmental modes contributing, then the signals could be the same even when the olfactant vibrations differ. See Section 5.2 for a further discussion of the effect of timescales.

Another interesting example within this category arises from the work done by Saito *et al.* [68] who measure mouse receptor level responses to a plethora of odorant stimuli. Contrasting similar-shaped 1-octanol and octanethiol for example it can be observed both activate the same number of receptors, but only 2 in common. Furthermore, they activate these receptors with varying EC50's. This demonstrates that similarly shaped odorants will smell different in character (according to “combinatorial coding” one receptor difference can alter the smell signal) but also likely different in odor thresholds [66]. This indicates that vibrational analysis may explain different receptor type activation whereas the differing EC50's may occur from the differing affinities of the O-H moiety versus the S-H present in the molecule.

### 4.6. Smell Alters with Increasing Concentration

The distinction between the affinity of an olfactant for a particular receptor and its efficacy, determined by the signals initiated to the brain, can be crucial. One underlying question is whether the receptors are binary, having only on and off states. Rhodopsin receptors are known to be binary, *i.e.,* on or off states only. But in the case of the *β*_2_- adrenergic receptor (one of the better characterized GPCRs), dopamine (a weak partial agonist) is just as efficacious as isoproterenol (full agonist) in disrupting what appears to be the molecular switch, but this is not enough to induce the full activation of the receptor as in the case of isoproterenol [69]. Further, it has been shown [69] using fluorescence resonance energy transfer (FRET) that, for the bimane-tryptophan quenching system, different types of agonists induce different types of conformational states, an observation which contradicts the binary proposition: the ligands do not simply modulate the equilibrium between an active on and inactive off state, but there are many degrees in between. Receptors are not always binary. But are olfactory receptors binary? In a swipe card model like the inelastic tunnelling model, there are still some important features that we cannot yet decide. When an odorant binds to a receptor, can more than one electron tunnel, limited only by electron supply to D or removal from A? Or does the odorant need to leave and be replaced before the next electron can contribute to the signal? The concentration dependence of odour therefore introduces an extra degree of complexity.

**Figure 11.**
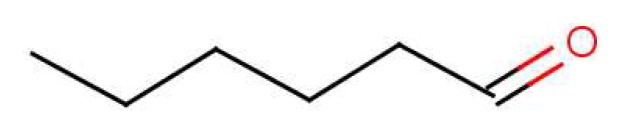
Hexanal, smell changes with increasing concentration.

If the olfactory receptors were binary, the potency of an odorant's signal could be directly attributed to the number of receptors occupied by odorant molecules. The potency would thus vary linearly with concentration, at least at low concentrations. In olfaction, this is notoriously not the case: in many cases, the higher the concentration, the more likely an odorant is to change its character [59,70,71], which implies at saturation certain “wrong” odorants are likely to find their way into an olfactory receptor and, whilst they may fit and bind inefficiently, they still activate the olfactory receptor to a *degree*. Smell change with increasing concentration suggests that, as absolute receptor saturation is approached, some odorants can activate non-parent receptors. Receptors that are unimportant at low concentrations become significant when some other receptors are saturated, see Figure 11.

### 4.7. Conformationally Mobile Enantiomers

Enantiomers—chiral molecules M with left- and right-handed mirror image forms—should all smell different in the simplest shape-based theories. More sophisticated (but less predictive) shape-based ideas argue that smell is combinatorial, and that parts of the odorant are detected by particular receptors ref 2; this is also known as the Odotope theory. Even so, it becomes hard to understand how enantiomers have different odours if only functional groups are detected by individual receptors; any chirality is lost and all enantiomers would smell the same. In the simplest frequency-based models, since left- and right-handed variants have exactly the same frequencies, all enantiomers should smell the same. However, as we have been emphasizing, other factors influence the response of the (chiral) olfactory receptors: it is not just the frequencies, but their couplings to the electron transition are important, and also the matrix element determining that transition. In a swipe card model, it is these extra factors that are critical in deciding whether chirality matters.

**Figure 12.**
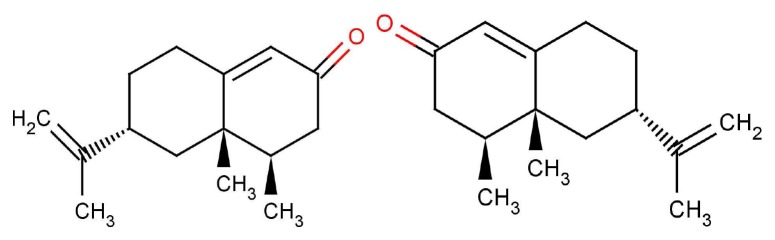
2 Nootkatones: the 4R, 4aS, 6R(+) enantiomer (left) smells of grapefruit (odor threshold 0.8 ppm) and its mirror is “woody, spicy” (threshold 600 ppm) [60]. Note also the (+)-enantiomer is around 750 times more potent odorant than the (−)-enantiomer [61].

Chiral molecules, as mirror images, see Figure 12, will have the same frequencies, and need the same *δ* in a receptor. However, in any given receptor, *t* and *S* will differ, since—as shown in the figure—the two cases are not superimposable. The issue of superimposability fits well with the general swipe card approach. We have shown previously [27] that these ideas can indeed predict whether enantiomers will be differentiated. These ideas suggest that chiral molecules will be distinguishable to some extent. Receptors are clearly very sensitive to structural variations, and any change in stereochemistry would affect actuation. Enantiomers have mirror image conformations that will asymmetrically activate the same chiral receptor, and any other conformational freedoms would exacerbate this.

Usually flexibility is said to aid receptor actuation, as if affinity where the only consideration. This seems not to be the case in olfaction, where conformational mobility can be either an aid or a hindrance in receptor actuation. It is assumed flexibility, in the sense of adapting to a binding pocket, is tantamount to agonistic behaviour. The phenomenon of flexible and distinguishable enantiomers however highlights the importance of efficacy and affinity *in combination* for actuation. This might be associated with features well known in non-radiative transition studies, but not normally considered in the biological context. For instance, we have concentrated on what, for non-radiative transitions, is the accepting mode; this takes up the energy in the non-radiative transition (and is the olfactant mode in our previous discussion). But other motions can affect the transition matrix element, and may enhance the transition; such modes are known as promoting modes. Promoting modes will have different symmetries, and may have substantial effects on *t*, again needing a more careful analysis than usually found.

### 4.8. Conformationally Immobile Enantiomers

**Figure 13.**
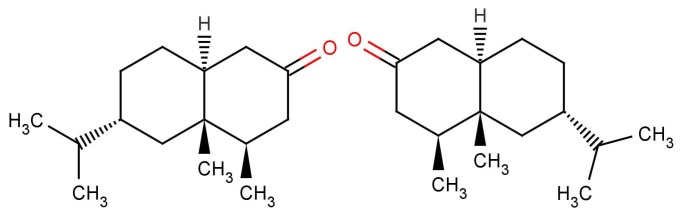
Type 1, tetrahydronootkatones smell “dusty-woody, fresh, green, sour, spicy, herbal, slightly fruity, animal, erogenic” on the left is (4R,4aS,6R,8aS)-(+)-tetrahydronootkatone and on the right its mirror image (4S,4aR,6S,8aR)-(−)-tetrahydronootkatone.

Whilst it is very rare that enantiomers smell exactly the same, both in intensity and in character, those that do share two common features. First, they have just one osmophoric group, a region of interesting electronegativity and superimposability. Secondly, they are not conformationally mobile. This is seen in the example in Figure 13, where any 6-membered ring flexibility is constrained [20]. One simple way to test for true type 1 (enantiomers that smell identical) is to smell a racemic mixture of the optical isomers. If the component parts are identical, then a mixture of the two must in turn be identical.

### 4.9. Steroids/pheromones

Why, in the case of 5*α*-androst-16-en-3-one, such as in Figure 1: can less than 100% of the population detect the naturally-occurring steroid? It is not difficult to believe that some people might miss one particular olfactory receptor, but smell is generally combinatorial, so would need a whole set of olfactory receptors to be missing [2]. However, as indicated earlier, pheromones are expected to behave differently from other olfactants with one receptor responding to one ligand through a lock and key mechanism. This is probably what is happening here. We note that detecting steroids would need a class of receptors with larger than average binding sites; steroids and hormone molecules have typically ∼55 atoms, whereas odorants are generally smaller, 3–20 atoms.

Thus, steroid and hormone receptors might work differently from those involved in smell (indeed, there may be crossover with the vomeronasal region).

## 5. Discussion

### 5.1. Donor and Acceptor Specifications

For an inelastic tunnelling mechanism to work, the molecular units D and A have to satisfy certain important constraints. Just what D and A are is not clear. They are probably common units among the likely receptor structures. They must be able to occur in two charge states, which we might call full and empty (so the transition takes D(full)A(empty) to D(empty)A(full)), though that is possible for many possible molecular units. Transition metals, often found in living systems, are among the species that can occur in several charge states. The D and A units must be able to revert back to their original states many times, *i.e*., D and A should not be destroyed in the olfaction process. It must be possible to feed an electron into D and remove an electron from A (*inter-* chain model) or return the electron to D (*intra-*chain model). To detect odorants within milliseconds, though tunneling via an odorant can be much faster, the replenishment of D and A should be within ms but not longer. Whilst that is not a strong constraint as regards timescale, it does require other reactions outside the receptor to maintain electrochemical equilibria that drive these motions. We note also that D and A must be sharp energy levels, which means only weak interactions to cause broadening. This is consistent with our calculated results, where all relevant interactions appear weak.

Perhaps the strongest constraint on D and A is the need for a small energy splitting *ε_D_* − *ε_A_* that corresponds to the small (but typical) vibrational quantum *ћω*_0_. Most olfactants M and many possible molecular units of the receptor are closed shell systems, and the gap between the highest occupied (HOMO) and lowest unoccupied (LUMO) levels is two orders of magnitude too large. Electron transfer from the HOMO of one unit to the LUMO of another is ruled out by their large energy difference, perhaps even 10 eV. One simple and general way round this problem is to assume that D and A are essentially the same molecular units, differing only slightly in geometry or because of slightly different units to which they are bound. We conjecture that likely donor/acceptor candidates are amino acid residues, perhaps of the same unit such as tryptophan (Trp). If, however, D and A are essentially identical (subject to minor differences already mentioned) for example two tryptophan residues (there are tryptophans that are highly conserved), we can imagine a suitably small splitting. As a hypothetical example, if D and A differed in energy solely because of single proton charge placed asymmetrically at 5Å from D and 4Å from A, this charge would the cause an energy separation $\frac{e^{2}}{ɛR_{A}}-\frac{e^{2}}{ɛR_{D}}\sim 0.72/ɛ\;\text{eV}$ which, for dielectric constant *ε* = 3, corresponds to 1935 cm^−1^.

Whilst lack of detailed receptor structural information means we cannot be too precise, it does make sense to suppose that D and A are typical units to be found in most—if not all—the receptor types, and that subtle modification by surrounding residues provides the fine tuning to different olfactant phonons. This would reconcile nicely with the observation that across OR types amino acids on helices 4 and 5 are highly variable (the moderating residues) and on helix 7 highly conserved (the staple residues D/A) [72]. We cannot be more precise without further experimental structural information and, in view of the considerable disagreements about odour receptors [73] we can make only very tentative observations. First, there are some common units, such as tryptophan, OH or SH groups, that might deviate in energy by small amount due to surrounding charge. Others have observed [74] that N-ethylmaleimide (NEM) reacts with the sulfhydryl groups in olfactory receptors rendering them irreversibly inactive; thus strongly suggesting that SH groups (perhaps in cysteine residues) might play a key role, possibly as D/A units. Secondly, there is evidence that very potent smelling odorants also bind strongly to zinc [8], and that a zinc deficiency results in anosmia reversible upon supplementing the diet. Conceivably Zn^2+^ or Cu^2+^ are components of electron donors, although it is possible their role involves protein structural stabilization as opposed to redox chemistry. The observation that zinc nanoparticles (but not zinc ions) can enhance the sensitivity of smell also suggests another role for zinc, perhaps as a source of electrons. Thirdly, recently the importance of NADPH towards GPCR functioning has been emphasized and investigated [75]; and also odorant binding proteins have a role not yet defined. One might conjecture that they are involved somehow in donating or recycling of electrons. Finally, we still do not know whether D and A are situated on two adjacent helices (*inter* helix tunnelling) or on a single helix (*intra* helix tunnelling, see Section 3.3). This raises the possibility that a bridge, like a disulphide bridge between two cysteine residues on one helix (with –S–S– and –SH HS– oxidation states), is a component of D or A, which has been postulated before [8].

For *inter*-helix tunnelling, we should ask what supplies the donor with its electron and removes it from the acceptor? We have assumed there is some electrochemical reaction or reactions that can achieve this, though it is perhaps not obvious in the olfactory biology what this source is. There are several possible explanations. One possibility is that odorant binding at the receptor site provides the energy and electrochemical requirements to prime D and A.

### 5.2. Timescales Depend on the Full System

The brain distinguishes odorants by using information from receptors. Communication is achieved via influxes of ions triggered by activated receptors, with the information somehow encoded as times between subsequent influxes. How does the brain distinguish between the influxes from activation of receptors by odorants from the occasional activation of receptors when they receive other molecules? There will be a small tunnelling rate even for an empty receptor, which presumably gives some background noise that the brain can filter out. But, in the inelastic tunnelling picture, are the tunnelling rates for the right odorant *M* (1/*τ_M_* say) and for the wrong odorant *W* (1/*τ_W_* say) sufficiently different? And how do these characteristic times *τ_M_*, *τ_W_* compare with the other times for steps in the overall process? We know, for instance, that odours can be detected in a time of perhaps a millisecond. Since this time involves the transfer of information from the receptor and interpretation in the brain, we should probably imagine events at the receptor itself taking perhaps a tenth of a millisecond.

One can imagine several different situations. One possibility is that the receptor itself inhibits signals from wrong molecules, perhaps because the molecule is resident for too short a time, or because there are competing processes we have yet to identify. Or, more generally, the brain could ignore signals below some threshold current, *i.e.*, less than some critical number of activations in a given interval. Thus Crick [76] discusses attentional mechanisms for vision, describing the possible “correlated firing” of neurons, and saying “spikes arriving at a neuron at the same time will produce a larger effect than the same number of spikes arriving at different times”. For olfaction, the spikes arriving at effectively the same time might correspond to a number of receptors activated in a period of less than or of the order of a millisecond. The inelastic tunnelling rates we calculated previously were much faster, corresponding to a characteristic time of the order of nanoseconds. Our earlier calculations suggested that even the characteristic times *τ_W_* for non-discriminating transitions were significantly shorter than a millisecond. We now offer several ways that this apparent contradiction can be resolved.

If the donor D and acceptor A could indeed be restored in times less than milliseconds, the shorter timescale for the right molecule (*τ_M_* ≪ *τ_W_*) would be reflected directly in more influxes during the period over which the neuron integrates producing a greater average current. That option seems more likely for *intra*-protein transfers (Figure 3) than for *inter*-protein transfers. If that were correct, the right molecule in a receptor could initiate several ionic influxes in each period of residence that become integrated into a single event by the brain.

Now suppose instead a receptor cannot send more than one signal in a ms, perhaps because of the slowness of the processes that ensure the donor D contains an electron and acceptor A is empty. Then both the right (discriminating) molecule *M* and wrong (non-discriminating) molecule *W* would cause a single influx in the integration time, and the brain would regard them as equivalent. Where might these assumptions be wrong? One possibility is that the tunnelling rates are really much slower, so only the right molecules *M* are effective, even on the millisecond timescale. A second possibility is that we need to examine not just the tunnelling event in isolation, but the whole sequence of events from the arrival of the electron at the donor to the docking and departure of the olfactant from the receptor.

Could the tunnelling rates be significantly less, yet still leave inelastic tunnelling a viable process? In our estimates, we used an extension of Marcus theory, involving a reorganization energy that is the first moment of the line shape function. This reorganization energy brings together all the couplings to modes at any one single frequency into a single mode by a linear transformation that is general for a harmonic system. In its usual form, there is the further assumption of a configuration coordinate that gathers modes of all frequencies into an effective environment coordinate with just one frequency. In the case of olfaction, as here, and other cases of very weak coupling, this second assumption is not essential and can be avoided. For olfaction, the important requirement is that the discrimination should not be limited by two phonon processes. With a one phonon process, there is the potential for the successful discriminating detection of odour. Two phonon processes and beyond introduce weaker, slower, signals that obscure discrimination. For such multiphonon processes, the modes can be those of the odorant or the environment. From the standard extension of Huang–Rhys theory [52] in the very weak coupling limit, the “right” transition has a probability proportional to the Huang–Rhys factor S_M_ for the discriminating mode. The competing “wrong” transition probability would be a two phonon transition, where two modes accept energy, and the corresponding factor is ½ S′S″, where S′ and S″ are the Huang–Rhys factors for these modes. Since values of S are in the range 0.01–0.3, this second probability could easily be smaller by a factor 100–500, or even more, since the phonon energy could be from environment modes that are less well coupled. Should the receptor be empty, the electronic transition matrix element would be reduced by a further factor. For example, a factor of order 30 (readily possible from simple models), would reduce the non-discriminating rate by a further factor of about 1000. So it seems possible that the non-discriminating transitions are weaker by a large enough margin to be at the level of noise.

We now consider the interplay between the electron transfer process and other processes to which it is coupled. There are two types we have investigated, both involving a race between two processes: in the first, there is a straight race between one process leading to signalling and another that frustrates it; in the second there is a race in which the competing process delays the signalling, but does not frustrate it.

In our model of the first type of interaction (the competing process frustrates signalling) we assume that, when the “right” odorant is in the receptor and when there is an electron in the donor D, then there is a constant probability that inelastic tunnelling occurs with a characteristic time *τ_M_* or *τ_W_*, depending on which molecule is present; as before, *τ_M_* ≪ *τ_W_*. We now also recognise that this key tunnelling process has competition. For instance, it might be prevented altogether if the electron on the donor D returns to the reservoir from which it came, or if the olfactant molecule leaves the receptor, or some further competing process. Suppose this competing process has a constant probability characterised by time *τ_R_*, largely independent of the odorant, but characteristic of the receptor and perhaps of the electronic reservoir that supplies electrons to D in the inter-protein case. In simple terms, there is a finite window of opportunity of order *τ_R_*. If this window is long enough for discriminating transitions (characteristic time *τ_M_*) then the odorant will indeed initiate a signal to the brain. If the time *τ_R_* is short enough relative to *τ_W_*, the characteristic time for non-discriminating transitions, then the wrong molecules will give signals only at the level of noise. We can readily calculate the ratio of successful odorant events leading to influxes for “right” molecules *M* and “wrong” molecules *W* as [*τ_R_* + *τ_W_*]/[*τ_R_* + *τ_M_*]. This ratio can be quite substantial: with a short window of opportunity, only the fast process will matter.

A description of our model of the second type of interaction (the competing process delays signalling) will be presented in a future publication. The main result is that the electron transition rates seen by the brain for both the “right” and “wrong” molecules get substantially reduced relative to the actual transition rates from D to A. This is because the electron can only reach A from D, but spends most of its time elsewhere. Thus, if the electron does not make a successful transition to A during one visit to D, the receptor has to wait for the return of the electron to D before another attempt is possible. The revised transition times are then $\tau_{M}^{\prime }=\tau_{D}(1+\tau_{M}/\tau_{R})$, and $\tau_{W}^{\prime }=\tau_{D}(1+\tau_{W}/\tau_{R})$, where *τ_D_* is the time associated with getting an electron to D, and might be much longer than the times needed to get an electron from D to A, and here *τ_R_* is the time taken for the electron to leave D by a competing route. Consequently the difference in time between signals $\left(\tau_{M}^{\prime }-\tau_{M}^{\prime }=(\tau_{M}-\tau_{W})\tau_{D}/\tau_{R}\right)$ could be much larger than the difference in the electron transfer times (*τ_M_* − *τ_W_*) if *τ_D_* ≫ *τ_R_*.

### 5.3. Summary

The development of the swipe card paradigm introduces a new and in many ways more satisfactory way of describing olfactory signal transduction. It gives a framework in which to evaluate critically theories like Turin's, and to identify key questions. Does a receptor measure a single electron crossing, (Figure 2) or several electrons crossing, or even none (Figure 3)? What is the nature of the olfactory G protein and how in turn does it propagate odorant dependent information? How many G-proteins are released? What are the turn-off mechanisms and the timescales olfaction may be limited by? What are good candidates for the donor and acceptor? Where does the supply of electrons come from? What happens to the odorant once it is smelled? Can we detect the odorant in a metabolized or even excited state?

There remains a wealth of opportunities for future research, notably experimental. The field is seriously limited by a lack of careful odorant physiological tests. Elimination of trace impurities is crucial (our noses can detect 1 part in a billion) as is the determination of non-subjective descriptors to describe odorant response. Compromise in these two considerations can lead to conflicting results [54,63]. To avoid dispute, olfaction and its dependence on odorant vibrational modes should be tested in double-blind tests with at least gas chromatograph pure samples. Only with fullest care could one answer with confidence whether humans detect isotopic changes. Simple racemic mixtures can test discrimination of enantiomers: odorants that truly smell the same will do so when mixed together. There are also plenty of more general non-transduction hypothesis laden experiments that can be conducted. To test for example more general olfactory receptor characteristics such as conformational changes: site-directed spin labelling, site-directed fluorescence quenching, sulfhydryl accessibility, disulphide cross-linking, spin labelling studies, an arsenal of techniques could be implemented to provide definitive answers. Questions could be: Are the receptors binary as in rhodopsin? Or do they possess many degrees of actuation as in recent discoveries [77] for the *β*2 adrenergic receptor? Given recent warnings on the amount of conflicting data analysis ubiquitous in olfaction (as it was for vision years ago) [73] we must be careful not to assume too much. The biophysical characterization of the olfactory receptors, is an integral next step to developing any theory. Most conclusions on the processes in olfactory signalling are based on sequence homology analysis that compares olfactory receptors to bovine rhodopsin, which, whilst helpful, may lead to assumptions that olfaction always works in similar ways. Yet the specialization of the olfactory class is still to be established; the OR's may be an entirely different GPCR class. Given the challenging cases above, they certainly seem to be overwhelmingly discerning. Given 50% of pharmaceuticals are targeted at GPCR's, there is not inconsiderable interest in this area [77] and the holy grail of X-ray crystallization and structural identification for olfactory receptors would mark considerable progress. That said, for serious modelling of D, A and tunnelling, positions of atoms to 0.1–0.2Å are needed, well beyond the best current data that resolves at best to 2Å. Since this is an order of magnitude too inaccurate for tunnelling rates, we may not be able to confirm too much, but there may at least be validation of what the predicted D/A units are. Further, the importance of a dynamic picture of the receptor is rapidly emerging [78,79], so the determination of a functioning coordinate frame would be a fundamental first step towards implementing molecular dynamics calculations to better understand the fluctuating world of receptor and ligand.

One criticism levelled against a non-standard theory of olfaction, like Turin's, is that nature reuses mechanisms that work, so one should expect many GPCRs to use electron transfer; this is not believed to be the case. However, even though many GPCRs might use electron transfer, it might not be an optimal solution in general. Olfaction is a special case in which receptors are most useful when promiscuous, *i.e*., an organism does not know in advance what chemical species it will encounter in its environment. Effectiveness in this situation is greatly aided by using molecular vibration frequencies to identify molecules, since it makes it possible to recognise small chemical groups from which molecules are composed. By contrast, many receptors are finely tuned to one or very few molecules, in which case shape alone may be superior. However, even when high chemical specificity is demanded, something additional to shape alone may be necessary: for example, it would appear that shape alone is inadequate for steroids.

## 6. Conclusions

### 6.1. The Future Prospects of the Turin Theory of Olfaction

We have given a critical analysis and review of a model for olfaction, attempting to address directly its main challenges. Alongside the shape-based lock and key ideas, the role of molecular vibrations has been around for many years, but only in 1996 was a specific mechanism for signal transduction proposed. Most of our present discussion of the swipe card model has concentrated on Turin's specific proposal that molecular vibrations provide the information for activation, and that the relevant vibration frequency is recognized by inelastic tunnelling. This proposal plus simple shape constraints, we believe, goes a long way towards understanding how odorants activate olfactory receptors. We have previously shown the ideas to be sound as regards physics, and need only realistic values of key parameters. In our present paper, we have chosen as many distinct examples as possible that confront this theory. To the extent that the data are good, Turin's proposal of vibration frequencies monitored by inelastic electron tunnelling stands up well. It cannot be the whole story, since such further factors as conformational mobility have a role. Nonetheless, the vibration frequency is a crucial part that can dominate smell, and the swipe card description appears to be a more useful paradigm than lock and key.

A shape-based theory cannot provide a full description of signal signatures, where the swipe card paradigm can. We know, for instance, that conformational mobility correlates with different odours for enantiomers [20]. We know that the receptor itself will be undergoing larger length-scale motions, as observed in protein studies. We can suspect that other dynamical aspects, from promoting modes to stochastic resonance, may have roles. Nonetheless, some form of inelastic electronic process seems a fully viable and important part of our sense of smell.

No theory of olfaction can hope to be comprehensive until at least two experimental developments have been achieved. First, there is a very clear need for further careful olfaction experiments, using at least gas chromatograph pure samples in double blind or similar quality tests. Secondly, we need a detailed structure of the olfactory receptor, good enough to define or dismiss particular ideas for the atomic-scale processes. There are many more questions to be answered, and the field invites many interesting experimental studies.

### 6.2. The Rise of Quantum Biology

One important consequence of going beyond discrimination based on shape alone is that quantum phenomena become much more evident. Shape, of course, already invokes implicitly the quantum nature of chemical bonding. Inelastic electron transitions of the sort discussed here involve a coherent quantum electron transfer event. Using vibrational frequencies as a discriminant relies on the quantum behaviour of the odorant vibrational modes, since energy can only be given to an oscillator in units of its vibrational quantum. There may be other quantum aspects, such as the role of zero-point motion, but these are not evident at this stage.

The lock and key paradigm was one of the earliest attempts to rationalize remarkably selective responses to different molecules. For large molecules, it is still a key concept. For small molecules, the underlying idea that shape is the sole critical factor fails badly. The swipe card paradigm, whether at this stage definitive as a model or not, introduces perhaps more productive ways of thinking that confront interesting observations in nature. For this reason alone it has the power to eliminate thinking based on theories that do not work and that road-block progress. Shape is not necessarily the actuating factor in smell; we must determine what factors are for reasons of phenomenological interest, but also because it is possible that the mechanisms and underlying processes of olfaction have parallels in the operation of a range of receptors activated by small molecules such as neurotransmitters, hormones, steroids, and so on. Since this is clearly a possible mechanism, surely nature and evolution would have used it somewhere! The details will never be precisely the same, but there is a clear grand challenge in the understanding of the responses of receptors to small molecules and linking them to their biomedical impacts.

## Figures and Tables

**Table 1.** Estimates for the several timescales for overall odorant recognition [20].

**Time Interval**	**Estimate**	**Description**
*T_X_*	10*μs* − 1*ms*	The time interval taken for reducing species X to diffuse through the cytoplasm.
*T_I_*	1*μs* − 1*ms*	The time taken for charge injection into a helical backbone of the protein.
*T_L_*	1*μs* − 1*ms*	The time taken for the charge to hop from RD on the helix to D (see Figure 2).
*τ_T_*_0_/*τ_T_*_1_	100*ns* − 0.1*ns*	The time taken for the electron to elastically or inelastically cross from D to A.
*T_R_*	1*μs* − 1*ms*	The time taken for the charge to hop from A on the helix to RA (see Figure 2).

**Table 2.** Estimated values for the parameters needed to compute *τ_T_*_0_ and *τ_T_*_1_ [20]. Note here we use *S* = 0.1, which is more realistic than our previous *S* = 0.01. We discuss below the likely sensitivities of the various parameter values, see section below

**Quantity**	*ћ****ω*_0_**	***S***	***λ***	|***t***|
Value	200 meV	0.1	30 meV	1 meV

**Table 3.** Interesting examples.

**Interesting cases**	**Examples**	**Observations**	**Conclusions**
*Isotopes*	Acetophenone & acetophenone-d8	*Drosophila melanogaster* and humans can differentiate these isotopes at better than chance odds [10,54]. Further drosophila can be *trained* to detect scent biased towards one isotope version.	This strongly indicates that vibrational modes are “smelt”. The notion that smell can be learnt (by training) indicates that there are innate abilities that are not employed unless it is necessary. Evolution has resulted in a diminishing sense of smell for humans where the sense is not as relied upon as it once was. This is indicated by the presence of 1,000 olfactory genes, only ∼390 of which are still functional, and 462 are pseudo-genes).

*Structurally similar odorants*	Sulphur compounds [i] *p*-menthene-1-en-8-thiol and its stereoisomer [ii]	[i] has an odour threshold of 10^−4^ppb and [ii] has an odour threshold of 10 ppb (100,000 times weaker) [55].	The two sulphur compounds will possess near identical partition coefficient in the mucus and likely reach the same receptors and have similar interactions at the binding site. This indicates the two molecules must differ in some other *action* at the site in order for there to be such a discrepancy in threshold. A possible alternative is that somehow after G-protein release the signal is amplified or reduced. But what feature of the odorant would tell the receptor to do this?
*Antagonists*	Eugenol (EG) and methyl isoeugenol (MIEG).	MIEG antagonizes the endogenous EG in a mouse receptor (mOR-EG). [56]. Further, undecanal antagonizes the endogenous bourgenol in human hOR17-4 [57].	Antagonism can occur at: the receptor level, the second messenger transduction level or at the membrane current level. In these studies ratiofluorometric studies were done to measure Ca^2+^influx and so indicate antagonism at the first step. Thus, olfactory receptors can be extremely sensitive and selective.

**Table 4.** Interesting examples continued.

**Interesting cases**	**Examples**	**Observations**	**Conclusions**
*Smell the same.*	Hydrogen sulphide and decaborane.	Hydrogen sulphide has the typical sulphuraceous smell and decaborane (though it contains no sulphur) also shares a “boiled onion, SH smell” [8].	Though no two odorants smell exactly the same (and have the same combinatorial code expressed to the glomeruli) there is some degree of overlap here. This poses the question: what causes two elementally and structurally very different molecules to activate some receptors in common?
*Smell different.*	Ferrocene and nickelocene.	Ferrocene smells spicy and nickelocene smells oily/chemical [8].	The only difference between these two examples is the metal ion in the centre of the structure. Something other than shape differentiates these two.
*Smell alters.*	Ambergris, hexanal.	Ambergris smells “oceanic” at low concentration and “Rotting” at high concentration [58]. Similar discrimination has also be seen at the receptor [2] and the glomerular level [59] for a range of other odorants.	At high enough concentrations receptors are recruited, where they otherwise would not be activated.

**Table 5.** Interesting examples continued.

**Interesting cases**	**Examples**	**Observations**	**Conclusions**
*Mobile enantiomers*	*(RSR*)-Nootkatone and (*SRS*)-nootkatone	The (4*R*, 4a*S*, 6*R)-*(+) enantiomer smells of grapefruit (0.8 ppm) and its mirror image smells “woody, spicy” (600 ppm) [60]. Note also the (+)-enantiomer is around 750× more potent than the (−)-enantiomer [61].	From a previous study [27] it has been found mirror image molecules with a 6-membered ring flexibility *always smell different* in their enantiomeric forms. This conformational mobility introduces an asymmetry where one hand is enabled to activate and the other hand is frustrated.
*Immobile enantiomers*	*(RSRS*)-Tetrahydronootkatone and (*SRSR*)-tetrahydronootkatone	Both smell “dusty-woody, fresh, green, sour, spicy, herbal, slightly fruity, animal, erogenic” [62]	From a previous study [27] it has been found mirror image molecules with a constrained 6-membered ring (a rigid molecule) *always smell the same* in their enantiomeric forms. This conformational rigidity reduces the asymmetry rendering these molecules superimposable from the receptor point of view.
*Steroids/pheromones*	5*α*-androst-16-en-3-one	5*α*-androst-16-en-3-one “smells strongly and disagreeably to about one third of people, one third smell it well, but do not describe it as particularly unpleasant; while one third cannot smell it” [55].	The “natural” steroid has a smell (according to a percentage of the population) implying *some* people do not have the equipment or ability to smell 5*α*-androst-16-en-3-one, or have not been trained to detect it.

## Appendix

### A. Simple Huang-Rhys Factor Model

The Huang-Rhys factor gives a measure of the coupling of the olfactant to the rapid movement of electron charge from donor D to acceptor A. We have done detailed calculations using density functional theory, and these will be reported in another paper. Here we describe the calculation for a simple model system, since this identifies some of the key dependencies in an analytical calculation.

Assume the olfactant is represented by a charge q bound harmonically to the centre of a cavity and able to move along the x axis. The harmonic vibration frequency is *ω*, and the force constant *K* = *Mω*^2^. We further assume that the electronic charge moves from one point D to another point A. We can choose these points to correspond to the *inter-*molecular (Figure 2) or *intra*-molecular (Figure 3) tunnelling cases. What we can estimate is the Huang-Rhys factor S as a function of the relative orientations of jump path and the oscillator axis, and also examine the dependence on the dipole moment and on the vibrational frequency.

The simplest calculation focuses on the change in the projection of the electric field along the oscillator axis as the electron moves from D to A. In Huang-Rhys, this transition corresponds to the switching on of a constant force that here we can represent as electric field having a projection *E* in the x direction. The new force is *F* = *Eq* on the oscillator charge. In consequence, the oscillator now starts moving about mean position *X* = *F*/*K* = *Eq*/*K*, so there is now a change in mean dipole *qX* = *Eq*^2^/*K*.

The relaxation energy that relevant for the Huang Rhys factor calculation is *R* = *F*^2^/2*K* = *E*^2^*q*^2^/2*K*. The Huang Rhys factor itself is *S* = *R*/*ћω* or (*E*^2^*q*^2^/2*K*)/*ћω*. Since the force constant *K* is *ω*^2^/*M* then *S* = *E*^2^*q*^2^/(2*Mћω*^3^). It then remains to calculate *E* for given positions of D and A, and also to decide what effective mass *M* and charge *q* is appropriate. This result is consistent with more formal theory of infrared absorption (*cf* Section 11.9.2 of [51]). The effective charges need careful discussion in any realistic case (see, e.g., [80]) but need not concern us so much in a model calculation.

When the oscillator is at the origin pointing along the x axis, we may take the donor D to be as at site *R⃗_D_* and the acceptor A at *R⃗_A_*. The change in force on transfer of an electron is

(5) $$F=\left(\frac{eq}{\in R_{D}^{3}}\right){\vec{R}}_{D}\cdot \vec{i}-\left(\frac{eq}{\in R_{A}^{3}}\right){\vec{R}}_{A}\cdot \vec{i}$$

with *i⃗* a unit vector along the x axis and ∈ the relevant dielectric constant. This can be evaluated easily in the model case. It is easily seen without explicit calculation that the relevant Huang Rhys factor falls off when the charges move away from the oscillator axis, or when the distances of D and A from the oscillator become more than a few Å.

The Huang-Rhys factors can be calculated using a full electronic structure code, and we shall describe this in detail elsewhere. This is normally done by combining total energies from four calculations; namely, for the system relaxed for the charge at *R⃗_D_*, calculate the energies when the electron is on D and when it is on A, and correspondingly, for the system relaxed for the charge at *R⃗_A_*, calculate the energies when the electron is on D and when it is on A. There is some redundancy in these calculations, but this compensates somewhat for working close to the limits of accuracy of electronic structure codes.

### B. The Electronic Matrix Elements

The important electronic states are those where the electron is on the donor, represented by the notation |*D*〉, and when the electron is on the acceptor, this is represented by the notation |*A*〉. We make the assumption that the electronic wavefunctions evolve adiabatically, as electron motion is very rapid compared with the nuclear motion during transitions. The Hamiltonian to describe these energetic states is:

(6) $$H_{e}=\left(\begin{array}{cc} {ɛ}_{D} & t\\ t & {ɛ}_{A} \end{array}\right)$$

where *t* is very small; the donor and acceptor are *weakly* coupled. If we introduce an odorant M into the equation then the electron can go from D to A via the molecule (or even as noted in the rest of the text, via a different route) with state |*M*〉. The Hamiltonian for this scenario is:

(7) $$H_{e}^{\prime }=\left(\begin{array}{ccc} {ɛ}_{D} & \upsilon & 0\\ \upsilon & {ɛ}_{M} & \upsilon \\ 0 & \upsilon & {ɛ}_{A} \end{array}\right)$$

In order to generalize to an effective two state Hamiltonian, we use downfolding:

(8) $$\left(\begin{array}{ccc} {ɛ}_{D} & \upsilon & 0\\ \upsilon & {ɛ}_{M} & \upsilon \\ 0 & \upsilon & {ɛ}_{A} \end{array}\right)\left(\begin{array}{c} c_{D}\\ c_{M}\\ c_{A} \end{array}\right)=ɛ\left(\begin{array}{c} c_{D}\\ c_{M}\\ c_{A} \end{array}\right)$$

This yields the secular equations:

(9) $$\begin{array}{c} {ɛ}_{D}c_{D}+\upsilon c_{M}=ɛc_{D}\\ \upsilon c_{D}+{ɛ}_{M}c_{M}+\upsilon c_{A}=ɛc_{M}\\ \upsilon c_{M}+{ɛ}_{A}c_{A}=ɛc_{A}. \end{array}$$

So *c_M_* = (*ε* − *ε_M_*) = *υ* (*c_A_* + *c_D_*), and $c_{M}=\frac{\upsilon }{ɛ-{ɛ}_{M}}(c_{A}+c_{D})$, so:

(10) $$\left(\begin{array}{cc} {ɛ}_{D}+\frac{\upsilon^{2}}{ɛ-{ɛ}_{M}} & \frac{\upsilon^{2}}{ɛ-{ɛ}_{M}}\\ \frac{\upsilon^{2}}{ɛ-{ɛ}_{M}} & {ɛ}_{A}+\frac{\upsilon^{2}}{ɛ-{ɛ}_{M}} \end{array}\right)\left(\begin{array}{c} c_{D}\\ c_{A} \end{array}\right)=ɛ\left(\begin{array}{c} c_{D}\\ c_{A} \end{array}\right)$$

This indicates a coupling $\frac{\upsilon^{2}}{ɛ-{ɛ}_{M}}=t\approx \frac{\upsilon^{2}}{{ɛ}_{D}-{ɛ}_{M}}$, from the off diagonal effective matrix elements. We approximate *ε* = *ε_D_* as we do not know the energy eigenstate *ε*, but assume *ε* = *ε_D_* or *ε_A_*, since *ε_D_* and *ε_A_* differ very little (meV), as compared with the difference between *ε_D_* and *ε_M_* (10's eV). Thus the initial electronic state will involve an admixture of D and M due to the presence of the odorant, and the final electronic state is [81] similarly an admixture of A and M. This implies the presence of an odorant M is integral to an electron transfer process.

### C. Table of Vibrational Frequencies

**Table 6.** A table of values from the AIST database, see http://riodb01.ibase.aist.go.jp/sdbs/cgi-bin/direct_frame_top.cgi, for the vibrational modes of ferrocene and nickelocene in cm^−1^.

**Ferrocene**	**Nickelocene**	**Ferrocene (cont.)**	**Nickelocene (cont.)**
3921	3926		1334
3443		1256	1259
3106	3127	1106	1110
3096	3095	1097	
3085	3082	1049	1046
	3072	1002	1003
	2973	866	880
2691	2661	849	839
2459		817	800
2264		788	772
1779	1774		659
1695	1677		650
1636	1670		640
	1663		620
	1571		486
	1554	493	
1410	1424	478	
